# Domestic Violence and Perinatal Mental Disorders: A Systematic Review and Meta-Analysis

**DOI:** 10.1371/journal.pmed.1001452

**Published:** 2013-05-28

**Authors:** Louise M. Howard, Sian Oram, Helen Galley, Kylee Trevillion, Gene Feder

**Affiliations:** 1 Section of Women's Mental Health, Institute of Psychiatry, King's College London, London, United Kingdom; 2 South London and Maudsley NHS Foundation Trust, Greater London, United Kingdom; 3 Academic Unit of Primary Health Care, School of Social and Community Medicine, University of Bristol, Bristol, United Kingdom; Massachusetts General Hospital, United States of America

## Abstract

**Background:**

Domestic violence in the perinatal period is associated with adverse obstetric outcomes, but evidence is limited on its association with perinatal mental disorders. We aimed to estimate the prevalence and odds of having experienced domestic violence among women with antenatal and postnatal mental disorders (depression and anxiety disorders including post-traumatic stress disorder [PTSD], eating disorders, and psychoses).

**Methods and Findings:**

We conducted a systematic review and meta-analysis (PROSPERO reference CRD42012002048). Data sources included searches of electronic databases (to 15 February 2013), hand searches, citation tracking, update of a review on victimisation and mental disorder, and expert recommendations. Included studies were peer-reviewed experimental or observational studies that reported on women aged 16 y or older, that assessed the prevalence and/or odds of having experienced domestic violence, and that assessed symptoms of perinatal mental disorder using a validated instrument. Two reviewers screened 1,125 full-text papers, extracted data, and independently appraised study quality. Odds ratios were pooled using meta-analysis.

Sixty-seven papers were included. Pooled estimates from longitudinal studies suggest a 3-fold increase in the odds of high levels of depressive symptoms in the postnatal period after having experienced partner violence during pregnancy (odds ratio 3.1, 95% CI 2.7–3.6). Increased odds of having experienced domestic violence among women with high levels of depressive, anxiety, and PTSD symptoms in the antenatal and postnatal periods were consistently reported in cross-sectional studies. No studies were identified on eating disorders or puerperal psychosis. Analyses were limited because of study heterogeneity and lack of data on baseline symptoms, preventing clear findings on causal directionality.

**Conclusions:**

High levels of symptoms of perinatal depression, anxiety, and PTSD are significantly associated with having experienced domestic violence. High-quality evidence is now needed on how maternity and mental health services should address domestic violence and improve health outcomes for women and their infants in the perinatal period.

*Please see later in the article for the Editors' Summary*

## Abstract 2

Louise Howard and colleagues conduct a systematic review and meta-analysis to estimate the prevalence and odds of experience of domestic violence experience among women with antenatal and postnatal mental health disorders.

*Please see later in the article for the Editors' Summary*

## Editors' Summary

**Background:**

Domestic violence—physical, sexual, or emotional abuse by an intimate partner or family member—is a major public health problem and although more common in women, can also affect men. Due to the nature of the problem, it is difficult to collect accurate figures on the scale of domestic violence, but a study by the World Health Organization in ten countries found that 15%–71% of women aged 15–49 years reported physical and/or sexual violence by an intimate partner at some point in their lives. Women experiencing domestic violence have significant short- and long-term health problems, particularly regarding their mental health: experience of domestic violence can lead to a range of mental health disorders such as depression, psychosis, eating disorders, and even suicide attempts.

**Why Was This Study Done?:**

As perinatal mental health disorders are among the commonest health problems in pregnancy and the postpartum period, and given the rate of domestic violence during pregnancy (previous studies have suggested a domestic violence prevalence of 4%–8% during pregnancy and the postnatal period), it is plausible that there may be a link between perinatal mental health disorders and having experienced domestic violence. Indeed, previous reviews have suggested the existence of such an association but were limited by the small number of included studies and focused on depression only, rather than the full range of antenatal and postnatal mental health disorders. So in this study the researchers systematically reviewed published studies to provide more robust estimates of the prevalence of having experienced domestic violence among women with antenatal and postnatal mental health disorders; the researchers also used a meta-analysis to estimate the odds (chance) of having experienced domestic violence among women with antenatal and postnatal mental health disorders.

**What Did the Researchers Do and Find?:**

The researchers searched multiple databases and hand searched three relevant journals using key search terms to identify all types of relevant studies. Using specific criteria, the researchers retrieved and assessed over 1,000 full papers, of which 67 met the criteria for their systematic review. The researchers assessed the quality of each selected study and included only those studies that used validated diagnostic instruments and screening tools to assess mental health disorders in their calculations of the pooled (combined) odds ratio (OR) through meta-analysis.

Using these methods, in cross-sectional studies (studies conducted at one point in time), the researchers found that women with probable depression in the antenatal period reported a high prevalence and increased odds of having experienced partner violence during their lifetime (OR = 3), during the past year (OR = 2.8), and during pregnancy (OR = 5). The results were similar for the postnatal period. The evidence was less robust for anxiety disorders: among women with probable anxiety in the antenatal period, the researchers found an OR of 2.9 of having experienced lifetime partner violence. The odds were less in the postnatal period (OR = 1.4) In their analysis of longitudinal studies (follow-up studies over a period of time), the researchers found an increased odds of probable postnatal depression both among women who reported having ever experienced partner violence in their lifetime (OR = 2.9) and among women who reported having experienced partner violence during pregnancy (OR = 3.1). The researchers also found a combined prevalence estimate of 12.7% for probable depression during the postnatal period following experiences of partner violence during pregnancy. Because of limited data, the researchers could not calculate an OR of the association between probable antenatal depression and later experiences of partner violence.

**What Do These Findings Mean?:**

These findings suggest that women with high levels of symptoms of perinatal mental health disorders—antenatal and postnatal anxiety, depression, and post-traumatic stress disorder—have a high prevalence and increased odds of having experienced domestic violence both over their lifetime and during pregnancy. However, these findings cannot prove causality, they fail to show a two-way association (that is, perinatal mental health disorders leading to subsequent domestic violence), and no information on other perinatal mental disorders, such as eating disorders and puerperal psychosis, was available. The variation of the quality of the included studies also limits the results, highlighting the need for high-quality data to suggest how maternity and mental health services could address domestic violence and improve health outcomes for women and their infants in the future. Nevertheless, this study emphasizes the importance of identifying and responding to possible domestic violence among women attending antenatal and mental health services.

**Additional Information:**

Please access these websites via the online version of this summary at http://dx.doi.org/10.1371/journal.pmed.1001452.

- The World Health Organization provides information and statistics about violence against women and also about mental health disorders during pregnancy
- The UK Royal College of Psychiatrists has information for professionals and patients about mental health disorders during pregnancy

## Introduction

Perinatal mental disorders are among the commonest health problems associated with pregnancy and the postpartum period. Antenatal disorders (including depression and anxiety disorders, eating disorders, and psychoses) are associated with adverse effects on the fetus including low birth weight and pre-term delivery [1]–[3], perinatal and infant death [4]–[6], and postnatal psychopathology [7]–[9], with the last associated with subsequent behavioural/emotional problems in the child and adolescent [10]. Risk factors for most perinatal mental disorders are generally similar to those for mental disorders outside the perinatal period and include a family and personal history of mental disorders [11],[12].

Previous research has found an association between mental disorder and being a victim of domestic violence (i.e., intimate partner violence and/or violence perpetrated by another family member) that is not diagnostically specific; associations have been found for common mental disorders, eating disorders, and psychosis and domestic violence in non-perinatal populations [13],[14]. The prevalence of domestic violence during pregnancy in high-income settings ranges from 4% to 8% in the majority of studies, equating to approximately 152,000 to 324,000 pregnant women experiencing abuse each year in the US [15],[16], in low- and middle-income countries the prevalence can be higher [17]. There is strong evidence that domestic violence increases the risk of low birth weight, and growing evidence of an association with pre-term labour, miscarriage, fetal death [18], and subsequent child behavioural problems [19]; domestic violence can also be a cause of maternal death [20]–[22]. The recent UK Confidential Enquiry into Maternal Deaths highlighted that domestic-violence-related deaths were perpetrated by both in-laws and partners [22].

Recent reviews have indicated a possible association between perinatal mental disorder and having experienced domestic violence, but these reviews have the following limitations: they identified only a limited number of relevant studies (<10); they focused predominantly on depression and not the full range of antenatal and postnatal mental disorders; they did not disaggregate findings according to whether violence was reported during pregnancy, during the past year, or over the lifetime; and they did not restrict their analyses to studies that used diagnostic or validated screening instruments to assess mental disorder [23]–[26]. Our systematic review aimed to address these limitations to provide more robust estimates of the following: (a) the prevalence of having experienced domestic violence (lifetime, past year, and during pregnancy) among women with antenatal and postnatal mental disorders (depression and anxiety disorders including post-traumatic stress disorder [PTSD], eating disorders, and psychoses including puerperal psychosis), (b) the odds of having experienced domestic violence (lifetime, past year, and during pregnancy) among women with antenatal and postnatal mental disorders (depression and anxiety disorders, eating disorders, and psychoses including puerperal psychosis), and (c) the odds of incident antenatal and postnatal mental disorders subsequent to having experienced domestic violence and the odds of experiencing domestic violence in women with pre-existing antenatal or postnatal disorders.

## Methods

### Search Strategy

This review followed MOOSE and PRISMA guidelines (see Text S1), and the protocol (see Text S2) is registered with the PROSPERO database of systematic reviews (http://www.crd.york.ac.uk/prospero; registration number CRD42011001281) [27],[28]. The search strategy comprised an electronic search of bibliographic databases, an update of a recent systematic review on the victimisation of people with mental disorders [29], hand searches of three key journals (*Trauma Violence and Abuse, Journal of Traumatic Stress*, and *Violence Against Women*), backwards and forwards citation tracking, and expert recommendations. Medical Subject Headings (MeSH) and text words were used to search 18 electronic databases, from their dates of inception up to 31 March 2011 (see Box 1 for the list of databases searched). Additional searches of Medline, Embase, and PsycINFO, and hand searches of *Trauma Violence and Abuse, Journal of Traumatic Stress*, and *Violence Against Women*, were conducted for the period 1 January 2011 to 15 February 2013. Terms for domestic violence were adapted from Cochrane protocols and peer-reviewed literature reviews, and terms for mental disorders were adapted from NICE guidelines [30]–[32]. The search strategy for Medline, Embase, and PsycINFO is shown in Text S3. When updating the victimisation review, we used the author's original search terms to search databases from September 2007 (the upper limit of the original review) to 31 March 2011. No language restrictions were used.

**Box 1**

**Biomedical databases**: Academic Search Complete, BNI (British Nursing Index), CINAHL (Cumulative Index to Nursing and Allied Health Literature), Cochrane, Embase, HMIC (Health Management Information Consortium), Medline, Maternity and Infant Care, PsycINFO, Science Direct, Web of Science (including SCI, SSCI, A&HCI, CPCI-S, CPCI-SSH).

**Social sciences databases**: Applied Social Sciences Index and Abstracts, International Bibliography of the Social Sciences, JSTOR, Sociological Abstracts.

**Theses and dissertations**: DART-Europe E-Theses Portal, EThOS, Networked Digital Library of Theses and Dissertations

### Selection Criteria

Studies were eligible for inclusion if they (a) included women who were 16 y or older and were assessed as having a perinatal mental disorder using a validated diagnostic instrument or screening instrument; (b) presented the results of peer-reviewed research based on experimental studies (e.g., randomised controlled trials, non-randomised controlled trials, parallel group studies), before-and-after studies, interrupted time series studies, cohort studies, case-control studies, or cross-sectional studies; and (c) measured the prevalence or odds of having experienced domestic violence during the lifetime, during the past year, (i.e., 12 mo prior to interview regardless of whether this was in the antenatal or postnatal period), or during pregnancy. When we identified multiple eligible papers from the same study, only the paper reporting the largest sample size was included.

### Data Extraction and Quality Appraisal

Two reviewers (S. O. and K. T.) screened the downloaded titles and abstracts against the inclusion criteria; if it was unclear whether a reference met the inclusion criteria, it was taken forward to the next stage of screening. Two reviewers (S. O. and K. T.) assessed the full texts of potentially eligible studies. If studies collected data on the prevalence and/or odds of domestic violence but did not report it, authors were contacted for further information.

Data from included papers were extracted into an electronic database by two reviewers (S. O. and H. G.). Extracted data included details of the study design, sample characteristics, measures of mental disorder and domestic violence, and the prevalence and odds of domestic violence victimisation. Details on the type of violence and chronicity of mental disorders were extracted where reported.

The quality of included studies was independently appraised by two reviewers (S. O. and H. G.) using criteria adapted from validated tools [33]. Reviewers compared scores and resolved disagreements before allocating a final appraisal score. The quality appraisal checklist (see Text S4) included items assessing study selection and measurement biases; studies were categorised as high quality if they scored ≥50% on questions pertaining to selection bias.

### Data Analysis

Prevalence, odds ratios (ORs), and 95% confidence intervals of having experienced domestic violence were calculated by type of perinatal mental disorder. If a study measured one disorder only (e.g., depression), the control group for the calculation of ORs was women without that disorder. If a study measured multiple disorders (e.g., depression and anxiety), the control group was women without those disorders. This reduced the risk of control groups including women with mental disorders, and improved consistency where studies contributed data for several mental disorders. Prevalence and unadjusted ORs were also calculated separately by period of violence experienced (lifetime, past year, and during pregnancy). We report prevalence estimates and ORs for having experienced “any violence” (i.e., any physical, sexual, or psychological violence). There were limited disaggregated data providing the prevalence and odds of having experienced physical, sexual, and psychological violence separately; these are reported in Table S1 (cross-sectional studies) and Table S2 (longitudinal studies).When analysing longitudinal data, we examined both the association between recent experiences of domestic violence at baseline and mental disorder identified at follow-up, and the association between mental disorder at baseline and domestic violence experienced during the follow-up period.

Pooled unadjusted OR estimates (with corresponding 95% confidence intervals) were calculated using random effects meta-analysis if data were available from three or more studies. We examined the influence of individual studies on summary effect estimates by conducting influence analyses, which compute summary estimates omitting one study at a time. We aimed to assess the risk of small study bias with funnel plots (see Figure S1) [34]. Because of the small number of eligible studies, statistical tests for funnel plot asymmetry were not appropriate, and we were confined to visual inspection of the plots. Heterogeneity among studies was estimated using the *I* ^2^ statistic. Pooled population attributable fraction (PAF) estimates were calculated using data from longitudinal studies, based on meta-analysis-derived summary relative risks. All analyses were conducted in Stata 11 [35].

Only studies that assessed mental disorders using either validated diagnostic instruments or validated screening instruments with the recommended cutoff scores were included in median prevalence and pooled OR calculations. Studies that used the Patient Health Questionnaire were excluded from these calculations because of the low sensitivity and specificity of the Patient Health Questionnaire in perinatal populations [36]. Where sufficient data were available, pooled ORs were also calculated that included only studies that used the Edinburgh Postnatal Depression Scale to measure probable depression (high levels of depressive symptoms), as this instrument is the most widely used internationally and has been validated in 32 languages [37].

## Results

**Figure 1:**
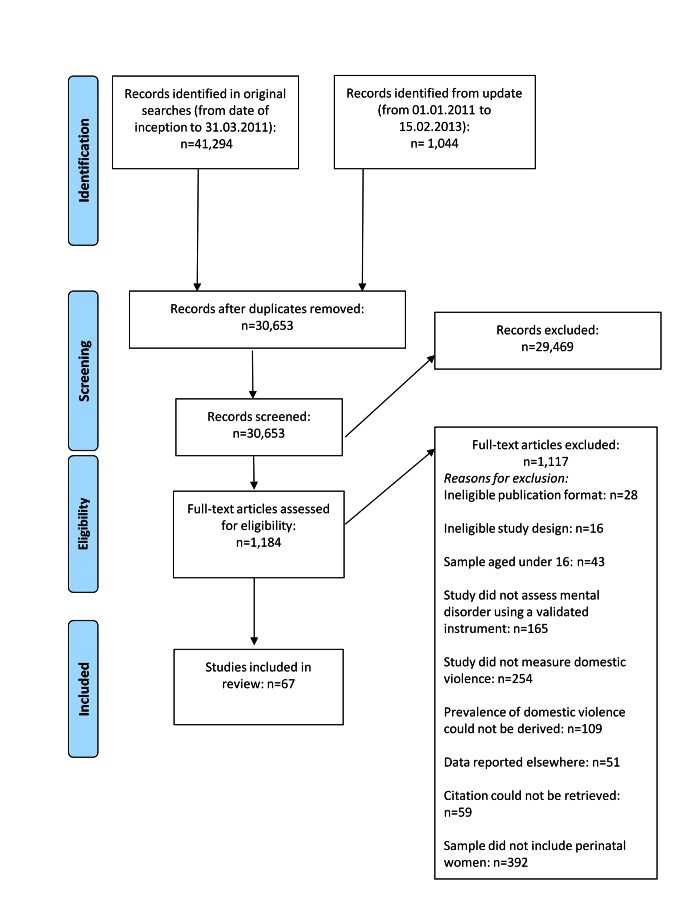
Flow diagram of screened and included papers.

The study selection process is presented in Figure 1. The literature search yielded 30,563 unique references, of which 29,469 were excluded following title and abstract screening. Of the 1,184 references that met, or potentially met, the inclusion criteria, 59 could not be located. Thus, 1,125 full papers were retrieved and assessed. Of these, 67 papers were included in the review following full-text screening; 55 were identified from searches of electronic databases, two from citation tracking, three from hand searching, and seven from expert recommendations.

### Key Features of Included Studies

A summary of included studies is shown in Table 1 [19],[38]–[103]. Individual details of all included studies, including outcomes and quality appraisal scores, are reported by disorder in Table S1 (cross-sectional data) and Table S2 (longitudinal data). Forty studies were categorised as high quality. Unless otherwise stated, the omission of individual studies during sensitivity analyses did not materially affect pooled ORs. Pooled ORs calculated using only studies that used the Edinburgh Postnatal Depression Scale were also not materially different to the pooled ORs calculated using all eligible studies, unless otherwise stated.

**Table 1:** Summary of included studies (*n* = 67).

Characteristic	Longitudinal Studies (*n* = 16)	Cross Sectional Studies (*n* = 56)	Total (*n* = 67)
**Diagnosis**			
Depression	16	45	56
Anxiety	1	4	5
PTSD	0	4	4
Psychological distress	0	10	10
**Recency of violence**			
Lifetime	7	25	31
Past year	5	22	25
During pregnancy	5	19	22
**Perpetrator of violence**			
Partner or spouse	16	56	67
Family member	1	8	9
**Type of violence**			
Physical	5	18	21
Psychological	3	15	17
Sexual	2	6	8
Physical, sexual, or psychological (combined)	10	41	48
**Setting**			
Clinical only	1	34	34
Non-clinical only	6	22	26
Clinical and non-clinical	9	0	9
**Region**			
North America	4	21	22
Central America	0	0	0
South America	2	7	9
Europe	2	6	7
Middle East	1	3	4
Africa	0	1	1
Asia	6	13	19
Australasia	1	5	5

Categories are not mutually exclusive, and row totals may therefore add to more than 67. Studies may contribute both longitudinal and cross-sectional data; column totals may therefore be less than the sum of the longitudinal and cross-sectional data columns.

### Findings from Cross-Sectional Data

**Figure 2:**
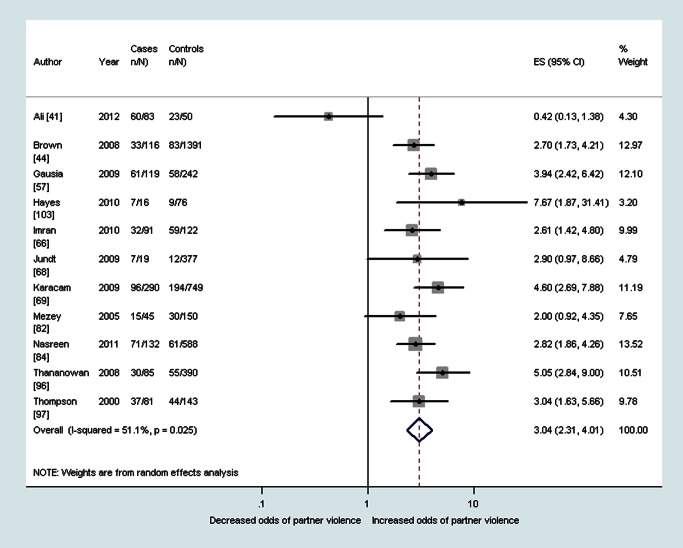
Meta-analysis of the association between antenatal depression and any lifetime domestic violence (cross-sectional studies). ES, effect size.

**Figure 3:**
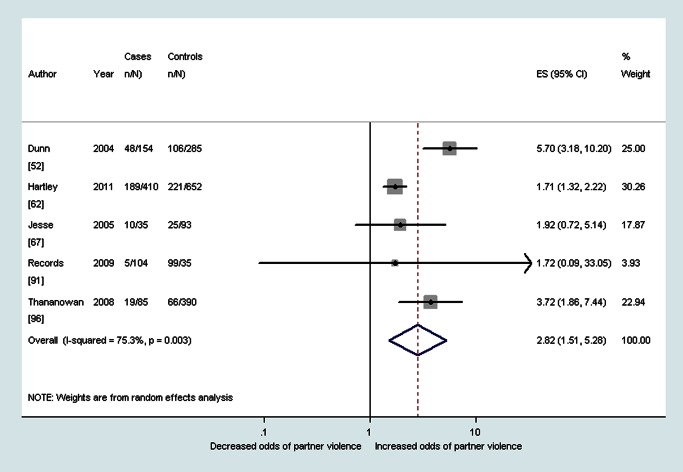
Meta-analysis of the association between antenatal depression and any past year partner violence (cross-sectional studies). ES, effect size.

**Figure 4:**
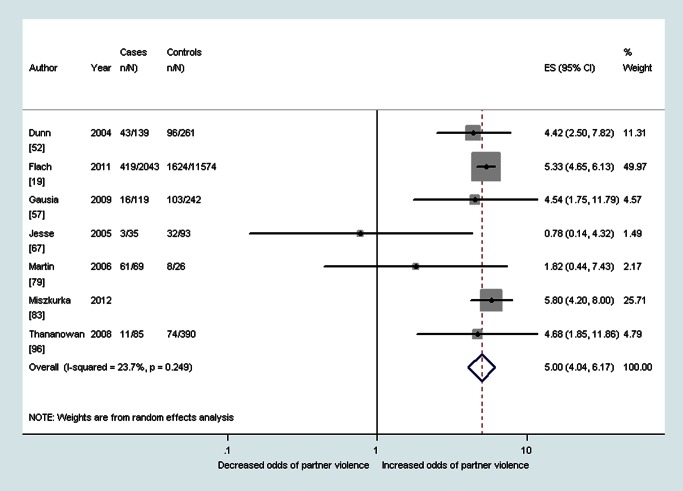
Meta-analysis of the association between antenatal depression and partner violence during pregnancy (cross-sectional studies). ES, effect size.

**Figure 5:**
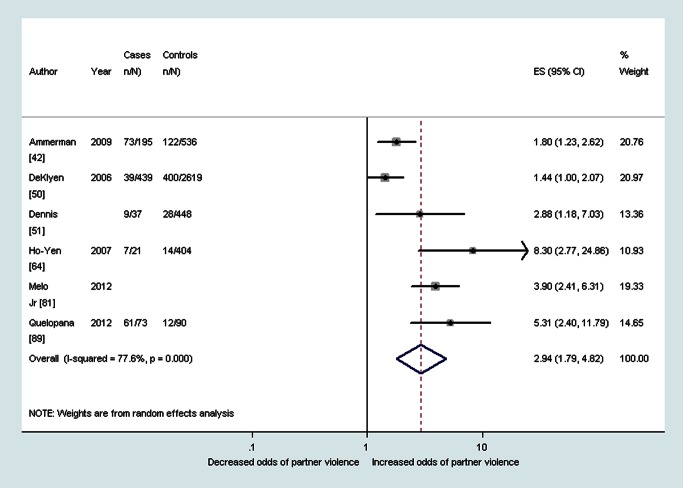
Meta-analysis of the association between postnatal depression and any lifetime partner violence (cross-sectional studies). ES, effect size.

**Figure 6:**
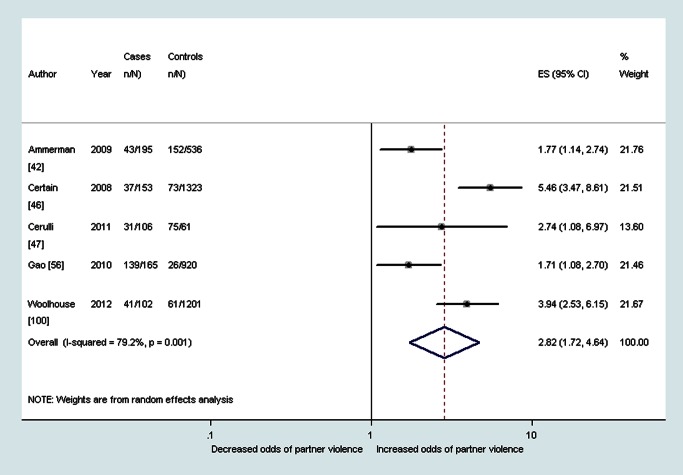
Meta-analysis of the association between postnatal depression and any past year partner violence (cross-sectional studies). ES, effect size.

**Figure 7:**
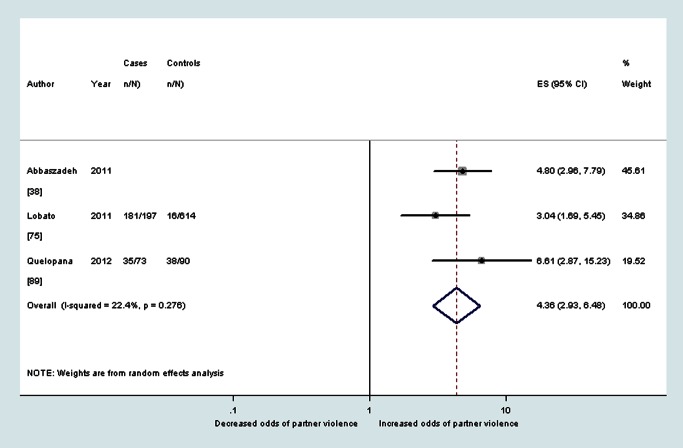
Meta-analysis of the association between postnatal depression and partner violence during pregnancy (cross-sectional studies). ES, effect size.

As shown in Table 2, median prevalence and pooled ORs showed that women with probable depression in the antenatal period reported a high prevalence and increased odds of having experienced intimate partner violence during the lifetime (OR 3.0, 95% CI 2.3–4.0, *I* ^2^ 51.1%), during the past year (OR 2.8, 95% CI 1.5–5.3, *I* ^2^ 75.3%), and during pregnancy (OR 5.0, 95% CI 4.0–6.2, *I* ^2^ 23.7%) (see also Figures 2–4). The heterogeneity for having experienced intimate partner violence during the lifetime was substantially reduced when omitting two studies that used the Hospital Anxiety and Depression Scale (OR 3.3, 95% CI 2.7–4.0, *I* ^2^ 11.2%). Median prevalence and pooled ORs also showed that women with probable depression in the postnatal period reported a high prevalence and increased odds of having experienced intimate partner violence during the lifetime (OR 2.9, 95% CI 1.8–4.8, *I* ^2^ 77.6%), during the past year (OR 2.8, 95% CI 1.7–4.6, *I* ^2^ 79.2%), and during pregnancy (OR 4.4, 95% CI 2.9–6.5, *I* ^2^ 22.4%) (see also Figures 5–7).

**Table 2:** Summary of findings from cross-sectional studies.

Disorder	Lifetime Domestic Violence			Past-Year Domestic Violence			Domestic Violence during Pregnancy		
	Median Prevalence	Pooled OR	Individual ORs Where Pooled OR Could Not Be Calculated	Median Prevalence	Pooled OR	Individual ORs Where Pooled OR Could Not Be Calculated	Median Prevalence	Pooled OR	Individual ORs Where Pooled OR Could Not Be Calculated
**Disorder in the antenatal period**									
Depression	38.9% (IQR 35.2%–51.3%, range 15.2%–72.2%) [41],[44],[57],[66],[68],[69],[82],[84],[92],[96],[97],[99],[103]	3.0 (95% CI 2.3–4.0, *I* ^2^ 51.1%) [41],[44],[57],[66],[68],[69],[82],[84],[96],[97],[103]	n/a	29.9% (IQR 21.8%–45.1%, range 4.8%–46.1%) [49],[52],[62],[63],[67],[71],[91],[96]	2.8 (95% CI 1.5–5.3, *I* ^2^ 75.3%) [52],[62],[67],[91],[96]	n/a	14.4% (IQR 13.2%–25.7%, range 8.6%–88.4%) [19],[44],[52],[57],[67],[79],[96]	5.0 (95% CI 4.0–6.2, *I* ^2^ 23.7%) [19],[52],[57],[67],[79],[83],[96]	n/a
Anxiety	29.8% (IQR 28.8%–53.1%, range 28.8%–53.1%) [41],[68],[99]	—	Ali et al. [41] (OR 0.5, 95% CI 0.2–1.4); Jundt et al. [68] (OR 2.1, 95% CI 1.1–4.0)	—	—	—	—	—	—
PTSD	—	—	Mezey et al. [82] (OR 6.4, 95% CI 1.7–26.4)	—	—	Seng et al. [95] (OR 4.6, 95% CI 2.5–8.5)	—	—	Seng et al. [95] (OR 6.0, 95% CI 1.4–29.2)
**Disorder in the postnatal period**									
Depression	28.5% (IQR 23.7%–36.3%, range 8.9%–83.6%) [39],[42],[50],[51],[64],[89]	2.9 (95% CI 1.8–4.8, *I* ^2^ 77.6%) [42],[50],[51],[64],[81],[89]	n/a	26.7% (IQR 20.1%–45.9%. range 5.7%–97.9%) [42],[43],[46],[47],[49],[56],[65],[100]	2.8 (95% CI 1.7–4.6, *I* ^2^ 79.2%) [42],[46],[47],[56],[100]	n/a	—	4.4 (95% CI 2.9–6.5, *I* ^2^ 22.4%) [38],[75],[89]	n/a
Anxiety	—	—	DeKlyen et al. [50] (OR 5.6, 95% CI 3.2–9.7)	—	—	—	—	—	—
PTSD	—	—	—	—	—	Cerulli et al. [47] (OR 4.6, 95% CI 1.1–18.4)	—	—	—

n/a, not applicable.

Two studies measured experiences of family violence (including violence from a partner) among women with probable depression in the antenatal period, reporting prevalence estimates of 35.2% and 38.9% [66],[99]. ORs could be calculated for only one study, which reported an increased odds of ever having experienced domestic violence (including from a partner) among women with probable depression in the antenatal period (OR 2.6, 95% CI 1.3–5.2) [66]. One study measured experiences of domestic violence (including violence from a partner) among women with probable depression in the postnatal period and found increased odds of having experienced past-year violence compared to women without probable depression (OR 2.9, 95% CI 1.5–5.7) [74].

Data were limited on the prevalence and odds of having experienced domestic violence among women with probable anxiety disorder or PTSD in either the antenatal or postnatal period. The prevalence of having experienced intimate partner violence during the lifetime was reported by two studies to be 27.8% and 29.8% among women with probable anxiety in the antenatal period [68],[99], and by one study to be 27.6% for women with diagnosed anxiety disorder in the postnatal period [50]. Individual studies reported non-significant increases in the odds of having experienced lifetime partner violence among women with probable anxiety in the antenatal period (OR 2.9, 95% CI 0.9–8.4) [68] and among women with anxiety disorder at 12 mo postpartum (OR 1.4, 95% CI 1.0–2.1) [50], compared to women with no anxiety [41]. Studies suggested that women with probable PTSD in the antenatal period had an increased risk of having experienced intimate partner violence during the lifetime (OR 6.4, 95% CI 1.7–26.4) [82], during the past year (OR 4.6, 95% CI 2.5–8.5) [92],[95], and during pregnancy 6.0 (95% CI 1.4–29.2) [92],[95]. Only one study measured experiences of intimate partner violence among women with PTSD in the postnatal period: Cerulli and colleagues reported increased odds of having experienced past-year intimate partner violence among women with PTSD (OR 4.6, 95% CI 1.1–18.4) and a prevalence of 41.2% [47].

One study measured having ever experienced domestic violence (including violence from a partner) among women with and without probable anxiety in the antenatal period [41]. The study, conducted in Pakistan, found no significant difference in the odds of having ever experienced violence between women with and without probable anxiety (OR 0.5, 95% CI 0.2–1.4); this lack of difference may be due to the very high levels of violence reported among women both with probable anxiety (76.4%) and without (86.0%) [41]. No studies measured violence perpetrated by family members among women with probable or diagnosed PTSD in the antenatal or postnatal period.

No studies were found for other disorders in the antenatal or postnatal period.

### Findings from Longitudinal Data

**Figure 8:**
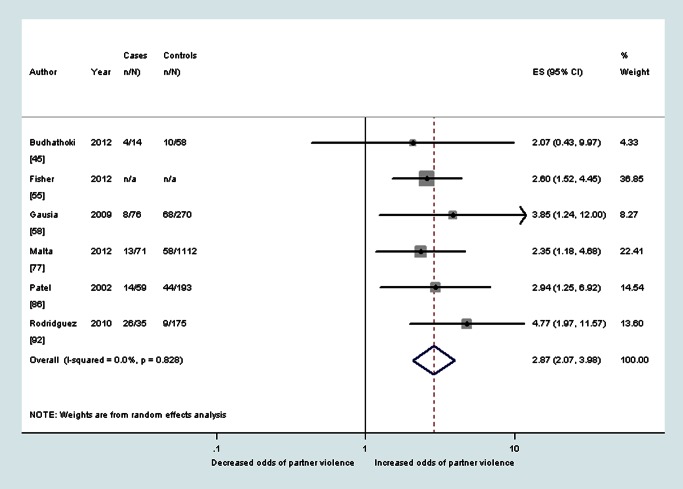
Meta-analysis of the association between any lifetime partner violence and postnatal depression (cohort studies). ES, effect size.

**Figure 9:**
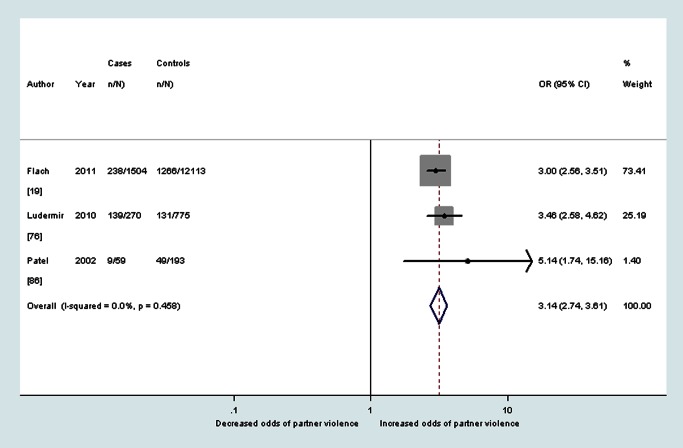
Meta-analysis of the association between any partner violence during pregnancy and postnatal depression (cohort studies).

Longitudinal data were collected by 16 studies. Twelve studies assessed the association between antenatal violence and later probable depression. Pooled ORs found increased odds of probable postnatal depression among women who reported at baseline having ever experienced intimate partner violence (OR 2.9, 95% CI 2.0–4.0, *I* ^2^ 0.0%) and among women who reported at baseline having experienced intimate partner violence during pregnancy (OR 3.1, 95% CI 2.7–3.6, *I* ^2^ 0.0%) (see also Figures 8 and 9). Neither estimate could be adjusted for antenatal depression because of a lack of data. The pooled PAF estimate for probable depression during the postnatal period following experiences of intimate partner violence during pregnancy was 12.7% (95% CI 11.8%–13.6%).

Five studies assessed the association between probable antenatal depression and later experiences of intimate partner violence. Pooled ORs could not be calculated because of insufficient data, but individual studies reported that the odds of having experienced intimate partner violence during or up to a year after pregnancy were between two and five times higher among women with probable depression in the antenatal period compared to women without probable depression (not adjusted for baseline violence). One cohort study reported increased odds of lifetime intimate partner violence reported at 4 mo postpartum among women who had probable antenatal anxiety at baseline (OR 1.7, 95% CI 1.1–2.7); no longitudinal data were available for other disorders.

## Discussion

### Main Findings

This systematic review and meta-analysis found that high levels of symptoms of all types of perinatal mental disorders included in studies to date (i.e., antenatal and postnatal anxiety, depression, and PTSD) were associated with having experienced domestic violence, although causality cannot be inferred. Pooled estimates from cross-sectional studies show that women with probable depression in the antenatal and postnatal periods have 3- to 5-fold increased unadjusted odds of having experienced domestic violence over the adulthood lifetime, during the past year, and during pregnancy, with correspondingly high prevalence estimates.

Meta-analysis of data from longitudinal studies suggests that women who experience domestic violence during pregnancy have 3-fold increased unadjusted odds of probable depression in the postnatal period. The pooled PAF of 12.7% (95% CI 11.8%–13.6%) calculated from these studies suggests that, if the association between domestic violence during pregnancy and postnatal depression are causal, experiences of domestic violence during pregnancy may contribute to the burden of postnatal mental disorder, and underlines the importance of domestic violence as a public health problem. Individual longitudinal studies also suggest that women with probable depression in the antenatal period have 3- to 5-fold increased odds of experiencing domestic violence during or up to a year after pregnancy. Although causality cannot be inferred, these findings suggest that a two-way association between experiences of domestic violence and probable depression in the antenatal and postnatal periods is likely, in which symptoms of depression may increase women's vulnerability to domestic violence, and having experienced domestic violence can increase the odds of probable depression in the antenatal and postnatal period. Insufficient data were available for other perinatal mental disorders to draw conclusions about the direction of causality for associations.

To our knowledge, this systematic review is the first to search for studies reporting on the prevalence and odds of having experienced domestic violence across the full range of antenatal and postnatal mental disorders. There are fewer studies on domestic violence and probable anxiety disorders than depression, but the review found, for the first time, consistent evidence of a high prevalence and increased odds of having experienced domestic violence among women with anxiety and PTSD in the antenatal and postnatal periods. We did not find any studies reporting the relationship between having experienced domestic violence and eating disorders or psychotic disorders, including puerperal psychosis, despite studies outside the perinatal period reporting an increased odds of having experienced domestic violence in women with eating disorders [14], and anecdotal reports of domestic violence associated with puerperal psychosis [104]. Further research is clearly needed for these diagnostic categories.

Most studies were carried out in high-income settings; findings were similar in low-income settings, but one study also reported that the odds of psychological distress associated with having experienced domestic violence was higher if the baby was a girl rather than a boy [86]. Risks are therefore likely to be modified by the cultural context of the pregnancy and postpartum period; this may be particularly the case where parents or parents-in-law play a major role in the postpartum period [22].

### Strengths and Limitations

Strengths of this review include restricting primary studies to those that used diagnostic instruments or validated screening instruments with their recommended cutoff scores to assess mental disorders. The comprehensive search strategy over multiple databases enabled the identification and synthesis of a large number of studies of several diagnostic categories, including depression, anxiety disorders, and PTSD. The review highlights critical gaps in the literature, including few longitudinal studies, few studies reporting on violence perpetrated by family members, and no studies investigating the possible relationship between domestic violence and puerperal psychosis.

There was high heterogeneity in pooled estimates of the association between having experienced past-year intimate partner violence and probable depression in both the antenatal and postnatal periods among cross-sectional studies, and there were insufficient studies to analyse the reasons for the higher heterogeneity using meta-regression. Visual inspection of the data, however, suggests that heterogeneity may be due to variation in the timing of recruitment, e.g., for women recruited in the last trimester, proportionally more of the “past year” reference period includes the time they were pregnant than for women recruited in the first trimester. Similarly, proportionally more of the “past year” reference period includes the period of pregnancy for women recruited in the early postpartum period than women recruited at 9–12 mo postpartum. This variation could be relevant because the prevalence of domestic violence can be lower during pregnancy [16],[17] and because the association between domestic violence and depression may vary as a function of when the violence occurred.

Insufficient characterisation of participants in the primary studies meant we were unable to assess the role of individual risk factors, such as social class. The lack of consistency in the type of data collected by the primary studies meant we were also unable to adjust estimates for potential confounders (e.g., history of depression or childhood abuse). In addition, most of the longitudinal studies did not provide data on baseline levels of symptoms or domestic violence, preventing clear interpretation on incident depression after domestic violence and vice versa. Thus, although having experienced domestic violence was strongly and consistently associated with probable antenatal and postnatal depression in both longitudinal and cross-sectional studies, we cannot draw firm conclusions about whether the observed association between domestic violence and probable perinatal depression is causal. As the calculation of the pooled PAF (the proportion of probable mental disorder potentially ascribable to exposure to intimate partner violence) is based on an assumption of causality, the PAF estimate should be treated with particular caution. Further high-quality longitudinal studies, including linked database studies, should be conducted to explore the nature of the association between domestic violence and perinatal mental disorder. Future research should also collect and report data on all types of violence (i.e., physical, sexual, and psychological violence); the majority (48/67) of the studies included in this review reported on physical violence—either alone or in combination with other forms of violence—and fewer than half reported prevalence and ORs disaggregated by type of violence.

### Implications

Domestic violence during pregnancy is associated with risks to the fetus, child, and mother [18]–[22]. Our finding that women with high levels of symptoms of a range of perinatal mental disorders have a high prevalence and increased odds of having experienced domestic violence both over the lifetime and during pregnancy highlights the importance of health professionals identifying and responding to domestic violence among women attending antenatal and mental health services. The World Health Organization and some international guidelines recommend identification of domestic violence and mental disorders in women attending antenatal care and mental health care [105]–[107]. However, a recent Cochrane review found little data on whether screening and other interventions improve outcomes for women experiencing domestic violence in the perinatal period [108]. Further data is therefore needed on how maternity and mental health services should best identify women with a history or current experience of domestic violence, respond appropriately and safely, and thus improve health outcomes for women and their infants in the perinatal period.

## Supporting Information

Figure S1 **Funnel plots to assess publication bias.**
(DOC)

Table S1 **Characteristics and reported outcomes of cross-sectional analyses of included studies.**
(DOCX)

Table S2 **Characteristics and reported outcomes of longitudinal analyses of included studies.**
(DOCX)

Text S1 **PRISMA checklist of items to include when reporting a systematic review or meta-analysis.**
(DOC)

Text S2 **Systematic review protocol.**
(DOC)

Text S3 **Search terms for Medline, Embase, and PsycINFO.**
(DOC)

Text S4 **Critical appraisal checklist for included studies.**
(DOC)

## Abbreviations

OR: odds ratio
PAF: population attributable fraction
PTSD: post-traumatic stress disorder
