# Supplementary material for: Domestic Violence and Perinatal Mental Disorders: A Systematic Review and Meta-Analysis
Source: PLoS Med. 2013 May 28;10(5):e1001452. doi: 10.1371/journal.pmed.1001452 (PMC3665851; doi:10.1371/journal.pmed.1001452)
Supplement: Figure S1 — Funnel plots to assess publication bias. (DOC) [file pmed.1001452.s001.doc]

**Figure S1: Funnel plots to assess publication bias**

**Figure 1: Funnel plot for odds of having experienced lifetime domestic violence among women with depression in the antenatal period (cross sectional studies)**

**
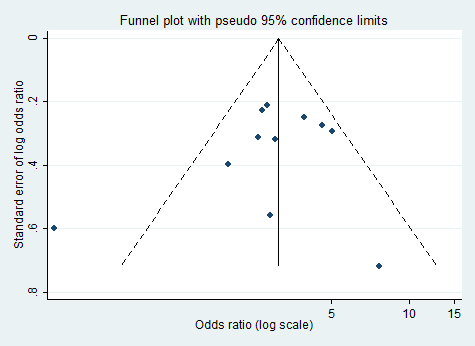
**

**Figure 2: Funnel plot for odds of having experienced past year domestic violence among women with depression in the antenatal period (cross sectional studies)**

**
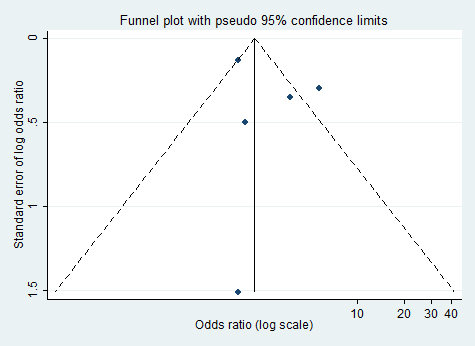
**

**Figure 3: Funnel plot for odds of having experienced domestic violence during pregnancy among women with depression in the antenatal period (cross sectional studies)**

**
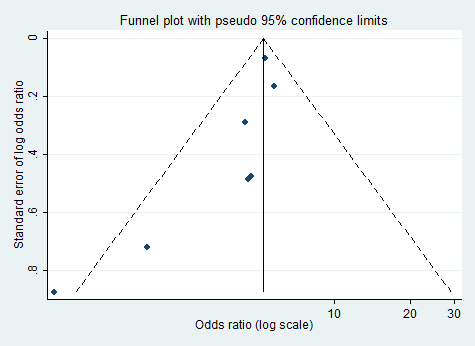
**

**Figure 4: Funnel plot for odds of having experienced lifetime domestic violence among women with depression in the postnatal period (cross sectional studies)**

**
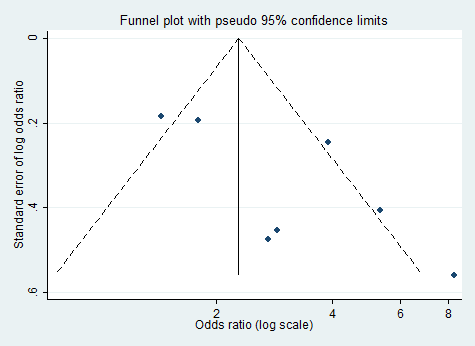
**

**Figure 5: Funnel plot for odds of having experienced past year domestic violence among women with depression in the postnatal period (cross sectional studies)**

**
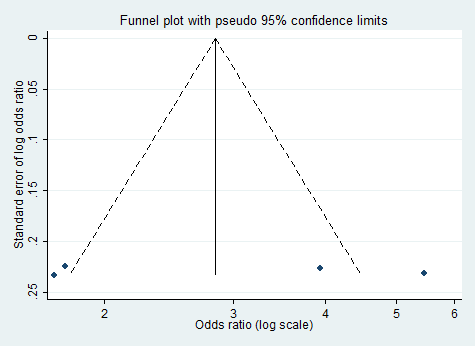
**

**Figure 6: Funnel plot for odds of having experienced domestic violence during pregnancy among women with depression in the postnatal period (cross sectional studies)**

**
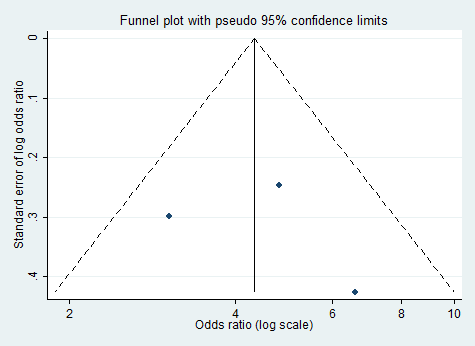
**

**Figure 7: Funnel plot for odds of having experienced lifetime domestic violence among women with depression in the postnatal period (cohort studies)**

**
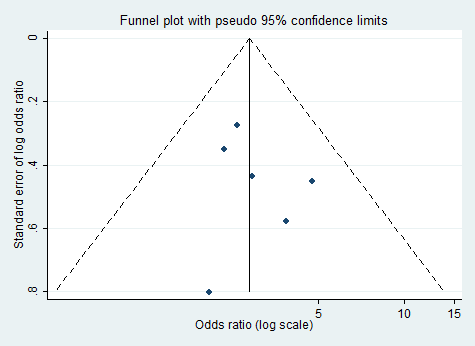
**

**Figure 8: Funnel plot for odds of having experienced domestic violence during pregnancy among women with depression in the postnatal period (cohort studies)**

**
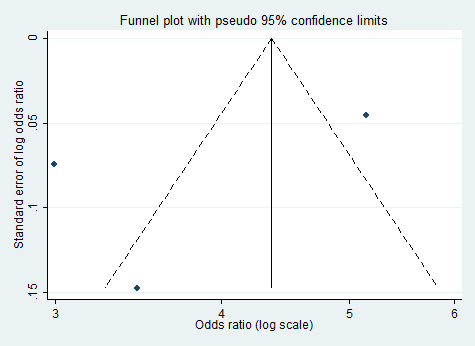
**
