# Supplementary material for: Domestic Violence and Perinatal Mental Disorders: A Systematic Review and Meta-Analysis
Source: PLoS Med. 2013 May 28;10(5):e1001452. doi: 10.1371/journal.pmed.1001452 (PMC3665851; doi:10.1371/journal.pmed.1001452)
Supplement: Table S1 — Characteristics and reported outcomes of cross-sectional analyses of included studies. (DOCX) [file pmed.1001452.s002.docx]

**Table S1: Characteristics and reported outcomes of cross-sectional analyses from included studies**

| **Author and year** | **Country** | | **Method** | **Sample size** | **Frequency of disorder** | **Type of domestic violence*** | **Prevalence & odds of DV** | **Quality appraisal score** | |
| --- | --- | --- | --- | --- | --- | --- | --- | --- | --- |
| **Antenatal Depression** | | | | | | | | | |
| Ali et al 2012[^41^](#_ENREF_67) | Pakistan | | Women attending antenatal clinics in a university hospital. Women interviewed during prenatal period.  Depression assessed using the Hospital Anxiety and Depression Scale (10 item, cut-off 8/10)  Lifetime physical or psychological partner or family violence assessed with questions developed by study authors. | 167 | With depression:  83/167 (49.7%)  Without depression:  50/167 (29.9%) | Lifetime:  132/167 (79.0%) | *Lifetime:*  With depression:  60/83 (72.2%)  Without depression:  43/50 (86.0%)  OR: 0.42 (0.1-1.1)  P=0.07 | Total score:  24/42  Selection quality score:  4/14  Measurement quality score:  13/14 | |
| Brown 2008.[^44^](#_ENREF_67) | Australia | | Women identified from hospital booking records and recruited by mail-out at <24 weeks gestation.  Depression assessed using the Edinburgh Postnatal Depression Scale (10 item, cut-off 12/13).  Lifetime partner violence and partner violence during pregnancy assessed using authors’ own questions about fearfulness. | 1507 | With depression:  116/1507 (7.7%)  Without depression:  1391/1507 (26.0%) | Lifetime:  232/1507 (15.4%)  During pregnancy:  47/1259 (3.7%) | *Lifetime:*  With depression:  33/116 (15.2%)  Without depression:  199/1391 (14.3%)  OR: 2.7 (1.7-4.2)  P<0.001  *Pregnancy*  With depression:  14/97 (14.4%)  Without depression:  33/1162 (2.8%)  OR: 5.8 (2.7-11.6)  P<0.001 | Total score:  30/42  Selection quality score:  11/14  Measurement quality score:  8/14 | |
| Cwikel 2003.[^49^](#_ENREF_68) | Israel | | Bedouin women recruited from health centre. Women interviewed during prenatal period.  Depression assessed using the Centre for Epidemiologic Studies Depression Scale (6 item, cut-off 4/5)  Past year partner violence assessed with questions taken from the Commonwealth Fund survey. | 25 | With depression:  4/25 (16.0%)  Without depression:  21/25 (84.0%) | Past year:  Any violence:  9/25 (36.0%)  Physical:  4/25 (16.0%)  Sexual:  1/25 (4.0%)  Psychological:  7/25 (28.0%) | *Past year*  *Any violence*  With depression:  2/4 (50.0%)  Without depression:  7/21 (33.3%)  OR: 2.0 (0.1-32.3)  P=0.54  *Physical*  With depression:  2/4 (50.0%)  Without depression:  2/21 (9.5%)  OR: 9.5 (0.4-186.1)  P=0.04  *Sexual*  With depression:  0/4 (0.0%)  Without depression:  1/21 (4.8%)  OR: n/a  *Psychological*  With depression:  2/4 (50.0%)  Without depression:  5/21 (23.8%)  OR: 3.2 (0.2-52.5)  P=0.29 | | Total score:  18/42  Selection quality score:  6/14  Measurement quality score:  6/14 |
| Dunn 2004.[^52^](#_ENREF_48) | USA | | Women recruited from prenatal clinics and interviewed at <14 weeks and >28 weeks gestation.  Depression assessed using the Centre for Epidemiologic Studies Depression Scale (10 item, cut-off 11/12).  Physical and psychological partner violence in the year prior to pregnancy and during pregnancy assessed using the Abuse Assessment Screen. | 439 | *1^st^ trimester*  With depression:  154/439 (35.1%)  Without depression:  285/439 (64.9%)  *3^rd^ trimester*  With depression:  139/400 (34.8%)  Without depression:  261/400 (65.2%) | *1^st^ trimester: past year:*  69/439 (15.7%)  *3^rd^ trimester: during pregnancy:*  67/400 (16.8%) | *1^st^ trimester: past year*  With depression:  48/154 (31.2%)  Without depression:  21/285 (7.4%)  OR: 5.7 (3.2-10.5)  P<0.001  *3^rd^ trimester: during pregnancy*  With depression:  43/139 (30.9%)  Without depression:  24/261 (9.2%)  OR: 4.4 (2.5-8.0)  P<0.001 | | Total score:  30/42  Selection quality score:  8/14  Measurement quality score:  13/14 |
| Flach 2011[^19^](#_ENREF_21) | UK | | Women recruited from during pregnancy as part of the Avon Longitudinal Study of Parents and Children and interviewed at 18 weeks gestation.  Depression assessed using the Edinburgh Postnatal Depression Scale (10 item, cut-off 12/13).  Physical and psychological partner violence during pregnancy assessed using authors’ own questions. | 13617 | With depression:  2043/13167 (15.0%)  Without depression:  11574/13167 (85.0%) | During pregnancy:  953/13617 (7.0%) | *During pregnancy:*  With depression:  419/2043 (20.5%)  Without depression:  534/11574 (4.6%)  OR: 5.3 (4.6-6.1)  P<0.001 | | Total score:  35/42  Selection quality score:  13/14  Measurement quality score:  12/14 |
| Gausia 2009.[^57^](#_ENREF_69) | Bangladesh | | Women sampled from primary healthcare clinic records and recruited and interviewed at home at 34-35 weeks gestation.  Depression assessed using the Bangla Edinburgh Postnatal Depression Scale (10 item, validated cut-off 9/10).  Lifetime and pregnancy physical marital violence assessed using authors’ own questions. | 361 | With depression:  119/361 (33.0%)  Without depression:  242/369 (67.0%) | Lifetime: 112/361 (31.0%)  During pregnancy: 22/361 (6.1%) | *Lifetime:*  With depression:  61/119 (51.3%)  Without depression: 51/242 (21.1%)  OR: 3.9 (2.4-6.5)  P<0.001  *During pregnancy:*  With depression:  16/119 (13.4%)  Without depression:  8/242 (3.3%)  OR: 4.5 (1.8-12.6)  P<0.001 | | Total score:  30/42  Selection quality score:  12/14  Measurement quality score:  11/14 |
| Gavin 2011.[^59^](#_ENREF_70) | USA | | Women recruited from a university-based delivery hospital and interviewed between 16-36 weeks gestation.  Depression assessed using the Patient Health Questionnaire (15 item, endorsement 5+ symptoms for more than half of days in last 2 weeks with at least 1 symptom being depressed mood or anhedonia).  Physical and sexual partner violence during pregnancy assessed using the Abuse Assessment Screen. | 2387 | With depression:  206/2387 (8.6%)  Without depression:  2181/2387 (91.4%) | During pregnancy:  76/2387 (3.2%) | *During pregnancy:*  With depression:  22/206 (10.8%)  Without depression:  54/2181 (2.5%)  OR: 4.7 (2.7-8.1)  P<0.001 | | Total score:  31/42  Selection quality score:  6/14  Measurement quality score:  13/14 |
| Hartley 2011[^62^](#_ENREF_71) | South Africa | | Women recruited from their households during the prenatal period.  Depression assessed using the Edinburgh Postnatal Depression Scale (10 item, cut-off 13/14, previously validated).  Past year physical partner violence assessed by author’s own questions. | 1062 | With depression:  410/1062 (38.6%)  Without depression:  652/1062 (61.4%) | Past year: 217/1062 (20.4%) | *Past year*  *Physical*  With depression:  189/410 (46.1%)  Without depression:  217/652 (33.3%)  OR: 1.7 (1.3-2.2)  P<0.001 | | Total score:  30/42  Selection quality score:  11/14  Measurement quality score:  9/14 |
| Harvey 2007.^63^ | Australia | | Audit of referrals from antenatal clinic to consultant liaison psychiatry service following screening at 10-12 weeks gestation.  Depression assessed using the Edinburgh Postnatal Depression Scale (10 item, cut-off 11/12).  No data available on assessment of past year domestic violence. | 102  Data on domestic violence missing for 17 women. | With depression: 102/102 (100.0%)  Without depression:  0/102 (0.0%) | Past year: 17/85 (20.0%) | *Past year*  With depression: 17/85 (20.0%)  Without depression: n/a  OR: n/a | | Total score:  31/42  Selection quality score:  8/14  Measurement quality score:  12/14 |
| Hayes 2010.^103^ | Australia | | Women recruited from an Aboriginal Medical Service antenatal clinic and interviewed during the prenatal period.  Depression assessed using the Edinburgh Postnatal Depression Scale (10 item, cut-off 12/13).  No information available on assessment of domestic violence. | 92 | With depression:  16/92(17.4%)  Without depression:  76/92 (82.6%) | Lifetime: 14/92 (15.2%) | *Lifetime*  With depression:  7/16 (43.8%)  Without depression:  7/76 (9.2%)  OR: 7.7 (1.8-32.0)  P<0.001 | | Total score:  29/42  Selection quality score:  5/14  Measurement quality score:  12/14 |
| Imran 2010.[^6^](#_ENREF_36)^6^ | Pakistan | | Women recruited from hospital antenatal clinics and interviewed during the third trimester.  Depression assessed using the Edinburgh Postnatal Depression Scale (10 item, cut-off 12/13).  Lifetime domestic violence assessed using authors’ own questions. | 213 | With depression:  91/213 (42.7%)  Without depression:  122/213 (57.3%) | Lifetime: 53/213 (24.9%) | *Lifetime*  With depression:  32/91(35.2%)  Without depression:  21/122 (17.2%)  OR: 2.6 (1.3-5.2)  P<0.001 | | Total score:  22/42  Selection quality score:  5/14  Measurement quality score:  8/14 |
| Jesse 2005.[^67^](#_ENREF_74) | USA | | Women recruited from and antenatal clinic and interviewed at 16-28 weeks gestation.  Depression assessed using the Beck Depression Inventory-II (21 item, cut-off 16/17).  Past year domestic violence and domestic violence during pregnancy assessed using the Abuse Assessment Screen. | 128 | With depression: 35/128 (27.3%)  Without depression: 93/128 (72.7%) | Past year: 26/128 (20.3%)  During pregnancy: 13/128 (10.2%) | *Past year*  With depression:  10/35 (28.6%)  Without depression:  16/93 (17.2%)  OR: 1.9 (0.7-5.2)  P=0.15  *During pregnancy*  With depression: 3/35 (8.6%)  Without depression: 10/93 (10.8%)  OR: 0.8 (0.1-3.3)  P= 0.72 | | Total score:  31/42  Selection quality score:  7/14  Measurement quality score: 12/14 |
| Jundt 2009.[^68^](#_ENREF_40) | Germany | | Women recruited from university hospital antenatal clinic and interviewed >24 weeks gestation.  Depression assessed using the Hospital Anxiety and Depression Scale (14 item, cut-off 8/9).  Lifetime physical and sexual marital violence assessed using the Abuse Assessment Screen. | 455 | With depression:  19/455 (4.2%)  With other disorder:  59/455 (13.0%)  Without disorder:  377/455 (82.9%) | Lifetime:  88/455 (19.3%) | *Lifetime*  With depression:  7/19 (36.8%)  Without disorder:  63/377 (16.7%)  OR: 2.9 (0.9-8.4)  P=0.02 | | Total score:  27/42  Selection quality score:  8/14  Measurement quality score:  8/14 |
| Karaçam 2009.^69^ | Turkey | | Women recruited from an antenatal clinic during the prenatal period.  Depression assessed using the Beck Depression Inventory (17 item, cut-off 16/17).  Lifetime physical, sexual and psychological domestic violence assessed using the authors’ own questions. | 1039 | With depression:  290/1039 (27.9%)  Without depression:  749/1039 (72.1%) | Lifetime:  Physical: 65/1039 (6.3%)  Sexual: 69/1039 (6.6%)  Psychological: 177/1039 (17.0%) | *Lifetime*  *Physical*  With depression:  40/290 (13.8%)  Without depression:  25/749 (3.3%)  OR: 4.6 (2.7-8.1)  P<0.001  *Sexual:*  With depression:  38/290 (13.1%)  Without depression:  31/749 (4.1%)  OR: 3.5 (2.1-6.0)  P<0.001  *Psychological:*  With depression:  96/290 (33.1%)  Without depression:  81/749 (10.8%)  OR: 4.1 (2.9-5.8)  P<0.001 | | Total score:  25/42  Selection quality score:  5/14  Measurement quality score:  10/14 |
| Kiely 2010.[^71^](#_ENREF_65) | USA | | Women recruited from community-based antenatal clinics serving mainly minority women and interviewed <28 weeks gestation.  Depression assessed using the Hopkins Symptom Checklist-Depression Scale (20 item, no information on cut-off)  Past year physical and sexual partner violence assessed using the Conflict Tactics Scale. | 1044 | With depression:  463/1044 (44.3%)  Without depression:  581/1044 (55.7%) | Past year: 336/1044 (32.2%) | *Past year*  With depression:  207/463 (44.7%)  Without depression:  129/581 (22.2%)  OR:2.8 (2.2-3.7)  P<0.001 | | Total score:  36/42  Selection quality score:  10/14  Measurement quality score:  14/14 |
| Martin 2006.^79^ | USA | | Women recruited from prenatal clinics serving predominantly low income women and recruited at 6-7 months gestation.  Depression assessed using the Centre for Epidemiologic Studies Depression Scale (20 item, cut-off of 15/16).  Physical, psychological and sexual partner violence during pregnancy assessed using the Conflict Tactics Scale-Revised. | 95 | With depression:  69/95 (72.6%)  Without depression:  26/95 (27.4%) | During pregnancy:  Physical:  38/95 (40.0%)  Sexual:  46/95 (48.4%)  Psychological:  82/95 (86.3%) | *During pregnancy:*  *Physical:*  With depression: 31/69 (44.9%)  Without depression: 7/26 (26.9%)  OR:2.2 (0.8-7.0)  P=0.11  *Sexual:*  With depression:37/69 (53.6%)  Without depression: 9/26 (34.6%)  OR: 2.2 (0.8-6.3)  P=0.10  *Psychological:*  With depression: 61/69 (88.4%)  Without depression: 21/26 (80.8%)  OR:1.8 (0.4-7.1)  P=0.33 | | Total score:  26/42  Selection quality score:  10/14  Measurement quality score:  10/14 |
| Mezey 2005.^82^ | UK | | Women recruited from hospital antenatal wards and interviewed during the prenatal period or at 1 day postpartum.  Depression assessed using the Edinburgh Postnatal Depression Scale (10 item, cut-off 12/13).  Lifetime physical or sexual partner violence assessed using the Abuse Assessment Screen. | 200 | With depression:  45/200 (22.5%)  Without disorder:  150/200 (75.0%) | Lifetime:  47/195 (24.1%) | *Lifetime*  With depression:  15/45 (33.3%)  Without disorder:  30/150 (20.0%)  OR: 2.0 (0.9-4.4)  P=0.06 | | Total score:  29/42  Selection quality score:  6/14  Measurement quality score:  11/14 |
| Miszkurka et al 2012^83^ | Canada | | Women recruited from four antenatal care clinics during the prenatal period.  Depression assessed using the Centre for Epidemiologic Studies Depression Scale (20 item, cut-off 15/16).  Physical, sexual and psychological partner violence assessed during pregnancy using an adapted version of the Abuse Assessment Screen. | 5,169 total sample:  3,768 Canadian-born women  1,401  immigrant women | *Total sample*  With depression:  1308/5169 (25.3%)  Without depression:  3861/5169 (74.6%)  *Canadian-born women*  With depression:  860/3768 (22.8%)  Without depression:  2908/3768 (77.2%)  *Immigrant women*  With depression:  448/1401 (32.0%)  Without depression:  953/1401 (68.0%) | During pregnancy:  *Total sample*  Any violence:  369/5169 (7.1%)  Physical:  172/5169 (3.3%)  Sexual:  31/5169 (0.6%)  Psychological:  220/5169 (4.3%)  *Canadian-born women*  Any violence:  277/3768 (7.3%)  Physical:  123/3768 (3.3%)  Sexual:  18/3768 (0.5%)  Psychological:  166/3768 (4.4%)  *Immigrant women*  Any violence:  92/1401 (6.6%)  Physical:  49/1401 (3.5%)  Sexual:  13/1401 (0.9%)  Psychological:  54/1401 (3.9%) | *During pregnancy (any violence):*  Total sample:  OR: 5.8 (4.2-8.1)  P<0.05  Canadian born women:  OR: 6.3 (4.25-9.39)  P<0.05  Immigrant women:  OR: 7.8 (4.4-14.1)  P<0.05 | | Total score:  26/42  Selection quality score:  5/14  Measurement quality score:  12/14 |
| Nasreen 2011^84^ | Bangladesh | | Women in the third trimester of pregnancy were recruited via the Bangladesh Rural Advancement Committee health programme registration.  Depression assessed using the Edinburgh Postnatal Depression Scale validated in Bangladesh (10 item, cut-off 9/10).  Lifetime physical and sexual partner violence was assessed using questions developed by the study authors. | 720 | With depression:  132/720  Without depression:  588/720 | *Lifetime:*  Physical:  243/720 (33.8%)  Sexual:  570/720 (79.2%) | *Lifetime:*  *Physical:*  With depression:  71/132 (53.8%)  Without depression:  172/588 (29.3%)  OR: 2.8 (1.9-4.2)  P<0.001  AOR: 1.69 (1.0-2.8)  P<0.05  *Sexual:*  With depression:  118/132 (89.4%)  Without depression:  452/588 (76.9%)  OR: 2.5 (1.4-4.9)  P<0.001  AOR: 1.95 (1.0-3.8)  P<0.05 | | Total score:  32/42  Selection quality score:  11/14  Measurement quality score:  12/14 |
| Nunes 2011^85^ | Brazil | | Women recruited from antenatal clinics and interviewed between 16 and 36 weeks gestation.  Depression assessed using the Brazilian Primary Care Evaluation of Mental Disorders depression module (9 items, cut-off 5/6).  Physical, sexual and psychological partner and family violence during pregnancy and lifetime partner violence assessed using the Abuse Assessment Screen. | 652 | With depression:  176/652 (27.0%)  Without depression:  476/652 (73.0%) | *Partner*  During pregnancy:  55/652 (8.4%)  Lifetime:  115/652 (17.6%)  *Family*  During pregnancy:  95/652 (14.6%) | *During pregnancy*  *Partner*  With depression:  35/176 (19.9%)  Without depression:  20/476 (4.2%)  OR:5.7 (3.1-10.7)  P<0.001  *During pregnancy*  *Family*  With depression:  55/176 (31.3%)  Without depression:  40/476 (8.4%)  OR:5.0 (3.1-8.0)  P<0.001  *Lifetime*  *Partner*  With depression  59/176 (33.5%)  Without depression:  56/476 (11.8%)  OR: 3.8 (2.4-5.9)  P<0.001 | | Total score:  30/42  Selection quality score:  7/14  Measurement quality score:  12/14 |
| Records  2009.^91^ | USA | | Women recruited from antenatal care clinics and interviewed in the third trimester.  Depression assessed using the Centre for Epidemiologic Studies Depression Scale (10 item, cut-off 15/16).  Past year partner violence assessed using the Severity of Violence Against Women Scale (cut-off 5/6). | 139 | With depression:  104/139 (74.8%)  Without depression:  35/139 (25.2%) | Past year:  6/139 (4.3%) | *Past year*  With depression:  5/104 (4.8%)  Without depression:  1/35 (2.9%)  OR: 1.7 (0.2-83.6)  P=0.62 | | Total score:  30/42  Selection quality score:  7/14  Measurement quality score:  13/14 |
| Rodriguez 2008.[^92^](#_ENREF_42) | USA | | Women recruited from antenatal clinics at 2 private healthcare organizations and interviewed at >12 weeks gestation.  Depression assessed using the Beck Depression Inventory Fast Screen (7 item, cut-off 3/4)  Lifetime partner violence assessed using the Abuse Assessment Screen. | 210 | With depression:  60/210 (28.6%)  Without disorder: data not available. | Lifetime:  92/210 (43.8%) | *Lifetime*  With depression:  38/60 (63.3%)  Without disorder: data not available.  OR: n/a | | Total score:  31/42  Selection quality score:  8/14  Measurement quality score:  11/14 |
| Thananowan 2008.[^96^](#_ENREF_78) | Thailand | | Women recruited from hospital antenatal clinics and interviewed during the prenatal period.  Depression assessed using the Thai Edinburgh Postnatal Depression Scale (8 item, non-validated cut-off 12/13).  Lifetime, past year and pregnancy physical, sexual and psychological partner violence assessed using the Abuse Assessment Screen. | 475 | With depression:  85/475 (17.9%)  Without depression:  390/475 (77.8%) | Lifetime:  68/475 (14.3%)  Past year:  47/475 (9.9%)  During pregnancy:  23/475 (4.8%) | *Lifetime:*  With depression:  30/85 (35.3%)  Without depression:  38/390 (9.7%)  OR: 5.1 (2.8-9.1)  P<0.001  *Past year:*  With depression:  19/85 (22.4%)  Without depression:  28/390 (7.2%)  OR: 3.7 (1.8-7.4)  P<0.001  *During pregnancy:*  With depression:  11/85 (12.9%)  Without depression:  12/390 (3.1%)  OR: 4.7 (1.8-12.0)  P<0.001 | | Total score:  27/42  Selection quality score:  5/14  Measurement quality score:  10/14 |
| Thompson 2000.[^97^](#_ENREF_79) | USA | | Women recruited from an antenatal clinic and interviewed during prenatal period.  Depression assessed using the Centre for Epidemiologic Studies Depression Scale (10 item, cut-off 15/16)  Lifetime partner violence assessed using the Abuse Assessment Screen. | 224 | With depression:  81/224 (36.2%)  Without depression:  143/224 (63.8%) | Lifetime:  68/224 (30.4%) | *Lifetime*  With depression:  37/81(45.7%)  Without depression:  31/143 (21.7%)  OR: 3.0 (1.6-5.7)  P<0.001 | | Total score:  32/42  Selection quality score:  9/14  Measurement quality score:  14/14 |
| Tuten 2004.^99^ | USA | | Substance abusing women recruited from the Center for Addiction and Pregnancy and interviewed during the prenatal period.  Depression assessed using the Structured Clinical Interview for DSM-IV.  Lifetime physical, sexual and psychological domestic violence assessed using the Women’s Psychosocial History questionnaire. | 102 | With depression:  18/102 (17.6%)  Without disorder: data not available. | Lifetime:  26/102 (25.4%) | *Lifetime*  With depression:  7/18 (38.9%)  Without disorder: data not available.  OR: n/a | | Total score:  24/42  Selection quality score:  4/14  Measurement quality score:  10/14 |
| **Antenatal anxiety** | | | | | | | | | |
| Ali et al 2012.^41^ | | Pakistan | Women attending antenatal clinics in a university hospital. Women interviewed during prenatal period.  Anxiety assessed using the Hospital Anxiety and Depression Scale (10 item, cut-off 8/10)  Lifetime physical or psychological partner or family violence assessed with questions developed by study authors. | 167 | With anxiety:  89/167 (53.3%)  Without anxiety:  50/167 (29.9%) | Lifetime:  132/167 (79.0%) | *Lifetime:*  With anxiety:  68/89 (76.4%)  Without anxiety:  43/50 (86.0%)  OR: 0.5 (0.2-1.4)  P=0.18 | Total score:  24/42  Selection quality score:  4/14  Measurement quality score:  13/14 | |
| Jundt 2009.[^68^](#_ENREF_40) | | Germany | Women recruited from university hospital antenatal clinic and interviewed >24 weeks gestation.  Anxiety assessed using the Hospital Anxiety and Depression Scale (14 item, no information on cut-off).  Lifetime physical and sexual marital violence assessed using the Abuse Assessment Screen. | 455 | With anxiety:  64/455 (14.0%)  With other disorder:  14/455 (3.1%)  With no disorder:  377/455 (82.9%) | Lifetime partner violence:  88/455 (19.3%) | *Lifetime*  With anxiety:  19/64 (29.8%)  Without any disorder:  63/377 (16.7%)  OR: 2.1 (1.1-4.0)  P=0.01 | Total score:  27/42  Selection quality score:  8/14  Measurement quality score:  8/14 | |
| Tuten 2004.[^99^](#_ENREF_37) | | USA | Substance abusing women recruited from the Center for Addiction and Pregnancy and interviewed during the prenatal period.  Anxiety assessed using the Structured Clinical Interview for DSM-IV.  Lifetime physical, sexual and psychological domestic violence assessed using the Women’s Psychosocial History | 102 | With anxiety:  18/102 (17.6%)  Without disorder:  data not available | Lifetime partner violence:  26/102 (25.4%) | *Lifetime*  With anxiety:  5/18 (27.8%)  Without any disorder:  data not available  OR: n/a | Total score:  24/42  Selection quality score:  4/14  Measurement quality score:  10/14 | |
| **Antenatal post-traumatic stress disorder** | | | | | | | | | |
| Mezey 2005.[^8^](#_ENREF_41)^2^ | | UK | Women recruited from hospital antenatal wards and interviewed during the prenatal period or at 1 day postpartum.  PTSD assessed using the Posttraumatic Diagnostic Scale (49 item, cut-off 17/18).  Lifetime physical or sexual partner violence assessed using the Abuse Assessment Screen. | 200 | With PTSD:  13/200 (6.5%)  Without disorder:  150/200 (75.0%) | Lifetime:  38/163 (23.3%) | *Lifetime*  With PTSD:  8/13 (61.5%)  Without disorder:  30/150 (20.0%)  OR: 6.4 (1.7-26.4)  P<0.001 | Total score:  29/42  Selection quality score:  6/14  Measurement quality score:  11/14 | |
| Rodriguez 2008.[^92^](#_ENREF_42) | | USA | PTSD assessed using the PTSD Checklist- Civilian Version (17 item, cut-off 44/45)  Lifetime partner violence assessed using the Abuse Assessment Screen. | 210 | With PTSD:  24/210  Without disorder: data not available. | Lifetime partner violence.  92/210 (43.8%) | *Lifetime*  With PTSD:  15/24 (62.5%)  Without disorder: data not available.  OR: n/a | Total score:  31/42  Selection quality score:  6/14  Measurement quality score:  14/14 | |
| Seng 2009.[^95^](#_ENREF_43) | | USA | Women recruited in maternity clinics (privately insured and Medicaid recipients) at <28 weeks gestation.  PTSD assessed using the National Women’s Study PTSD Module. Women who experienced trauma assessed as having PTSD if they simultaneously endorsed 1 re-experiencing symptom, 3 avoidance symptoms and 2 arousal symptoms, if the duration of disturbance is more than 1 month and the disturbance causes clinically significant impairment or distress.  Past year physical and sexual partner violence assessed using the Abuse Assessment Screen. | 1581 | With PTSD:  319/1581 (23.2%)  Without PTSD  1262/1581 (79.8%) | Past year partner violence:  50/1581 (3.2%)  During pregnancy:  10/1581 (0.6%) | *Past year:*  With PTSD:  26/319 (8.2%)  Without PTSD:  24/1262 (1.9%)  OR: 4.6 (2.5-8.5)  P<0.001  *Pregnancy:*  With PTSD:  6/319 (1.9%)  Without PTSD:  4/1262 (0.3%)  OR: 6.0 (1.4-29.2)  P=0.002 | Total score:  34/42  Selection quality score:  10/14  Measurement quality score:  14/14 | |
| **Antenatal psychological distress** | | | | | | | | | |
| Crempien 2011^48^ | | Chile | Women recruited from antenatal clinics and interviewed during pregnancy.  Psychological distress assessed using the General Health Questionnaire-12 (12 item, cut-off of 5/6).  Physical and psychological violence during pregnancy by a partner or family member assessed using a modified version of the Abuse Assessment Screen. | 256 | With psychological distress:  108/256 (42.2%)  Without psychological distress:  148/256 (57.8%) | During pregnancy:  *Physical:*  15/256 (5.9%)  *Psychological:*  77/256 (30.1%) | During pregnancy:  *Physical:*  With distress:  7/108 (6.5%)  Without distress:  8/148 (5.4%)  OR: 1.2 (0.4-4.0)  P=0.72  *Psychological:*  With distress:  49/108 (45.4%)  Without distress:  28/148 (18.9%)  OR: 3.6 (2.0-6.5)  P<0.001 | Total score:  20/42  Selection quality score:  5/14  Measurement quality score:  7/14 | |
| Ferrari-Audi 2008.[^53^](#_ENREF_80) | | Brazil | Women recruited from antenatal clinics and interviewed during the prenatal period.  Psychological distress assessed using the SRQ-20.  Lifetime physical, sexual and psychological partner violence assessed using a previously validated questionnaire. | 1379 | With psychological distress: 723/1379 (52.4%)  Without psychological distress: 656/1379 (47.6%) | Lifetime:  *Physical/sexual:*  89/1379 (6.5%)  *Psychological:*  263/1379 (19.1%) | *Lifetime*  *Physical/sexual:*  With distress: 72/723 (10.0%)  Without distress: 17/656 (2.6%)  OR: 4.2 (2.4-7.6)  P<0.001  *Psychological:*  With distress: 178/723 (24.6%)  Without distress: 85/656 (12.9%)  OR: 2.2 (1.6-3.0)  P<0.001 | Total score:  24/42  Selection quality score:  4/14  Measurement quality score:  12/14 | |
| Groves 2011^61^ | | South Africa | Women who were pregnant were recruited from a primary health care clinic  Emotional distress was assessed using the Hopkins Symptom Checklist (25 items (each rated on a point between 1 and 4), cut-off 44/100)  Physical, psychological and sexual partner violence during pregnancy was assessed using an adapted version of the World Health Organisation Violence Against Women instrument | 1402 | With emotional distress:  506/1402 (33.4%)  Without emotional distress:  896/1402 (63.9%) | *During pregnancy:*  Physical:  142/1402 (10.1%)  Psychological:  269/1402 (19.2%)  Sexual:  39/1402 (2.8%) | *During pregnancy*  Physical:  OR: 1.48 (1.28-1.71)  P<0.001  *AOR: 1.17 (0.99-1.38)  P=0.06  Psychological:  OR: 1.53 (1.38-1.69)  P<0.001  *AOR: 1.41 (1.26-1.57)  P<0.001  Sexual:  OR: 2.99 (1.74-5.13)  P<0.001  *AOR: 2.01 (1.16-3.77)  P=0.01  *(adjusted for marital status, gestational age, pregnancy intention, socio-economic status, relational control, childhood sexual abuse and social support) | Total score:  28/42  Selection quality score:  8/14  Measurement quality score:  11/14 | |
| Karmaliani 2009.[^70^](#_ENREF_81) | | Pakistan | Women recruited by health visitors during routine antenatal home visits and interviewed at 20-26 weeks gestation.  Anxiety/depression assessed using the Aga Khan University Anxiety and Depression Scale-Short Form (13 item, cut-off 12/13).  Physical and psychological domestic violence in the 6 months prior to pregnancy assessed using authors’ own questions. | 1368 | With anxiety or depression:  246/1368 (18.0%)  Without anxiety or depression:  1122/1368 (92.0%) | 6 months prior to pregnancy:  *Any violence:*  661/1368 (48.3%)  *Physical:*  208/1368 (15.2%)  *Psychological*:  408/1361 (30.0%) | *6 months prior to pregnancy:*  *Any violence*:  With distress:  189/246 (74.9%)  Without distress:  472/1122 (42.1%)  OR: 4.6 (3.3-6.4)  P<0.001  *Physical:*  With distress:  87/246 (35.4%)  Without distress:  121/1122 (10.8%)  OR: 4.5 (3.2-6.3)  P<0.001  *Psychological:*  With distress:  94/246 (38.2%)  Without distress:  314/1122 (28.0%)  OR: 1.6 (1.2-2.1)  P=0.002 | Total score:  31/42  Selection quality score:  10/14  Measurement quality score:  11/14 | |
| Kim 2006.[^72^](#_ENREF_82) | | USA | Women recruited from public hospital antenatal clinics and interviewed during the prenatal period.  Antenatal psychological distress assessed using the Primary Care Evaluation of Mental Disorders Patient Health Questionnaire.  No information on assessment of domestic violence. | 154 | With psychological distress:  48/154 (31.2%)  Without psychological distress:  106/154 (68.8%) | Past year domestic violence:  7/154 (4.5%) | *Past year*  With distress:  5/48 (10.4%)  Without distress:  2/106 (1.9%)  OR:6.1 (0.9-64.9)  P=0.02 | Total score:  24/42  Selection quality score:  5/14  Measurement quality score:  8/14 | |
| Patel 2002.[^86^](#_ENREF_54) | | India | Women interviewed at >30 weeks gestation.  Marital violence (lifetime and during pregnancy) assessed using author’s own questions.  Psychological distress assessed using the General Health Questionnaire-12 (12 item, cut-off of 5/6). | 270 | With psychological distress:  113/270 (41.9%)  Without psychological distress:  157/270 (58.1%) | Lifetime:  31/270 (11.5%)  During pregnancy: 15/270 (5.6%) | *Lifetime:*  With distress: 11/82 (13.4%)  Without distress: 20/153 (13.1%)  OR: 0.9 (0.4-2.1)  P=0.74  *During pregnancy:*  With distress: 7/82 (8.5%)  Without distress: 8/153 (5.23%)  OR: 1.7 (0.5-5.6)  P=0.32 | Total quality:  23/42  Selection quality:5/14  Measurement quality: 10/14 | |
| **Postnatal depression** | | | | | | | | | |
| Abbaszadeh 2011^38^ | | Iran | Women recruited in pediatric clinics and interviewed at 8 weeks postpartum.  Depression assessed using the Persian Edinburgh Postnatal Depression Scale (10 item, validated cut-off 12/13).  Physical and psychological domestic violence during pregnancy assessed using the Abuse Assessment Screen. | 400 | With depression:  183/400 (45.8%)  Without depression:  217/400 (54.2%) | During pregnancy:  262/400 (65.6%) | With depression:  Data not available  Without depression:  Data not available  OR:4.8 (2.9-7.8)  P<0.001 | Total score:  23/42  Selection quality score:  6/14  Measurement quality score:  /14 | |
| Ahmed 2012^39^ | | Iraq | Women recruited in antenatal clinics within primary health centers and interviewed at 6-8 weeks postpartum.  Depression assessed using a Kurdish translation of the Edinburgh Postnatal Depression Scale (10 item, non-validated cut-off 9/10).  Marital physical, sexual and threatened violence assessed using authors’ own questions. | 1000 | With depression:  284/1000 (28.4%)  Without depression: 716/1000 (71.6%) | Lifetime:  118/1000 (11.8%) | With depression:  67/284 (23.6%)  Without depression:  51/716 (7.1%)  OR: 4.0 (2.7-6.1)  P<0.001 | Total score:  21/42  Selection quality score:  6/14  Measurement quality score:  9/14 | |
| Ammerman 2009.[^42^](#_ENREF_83) | | USA | Women recruited through their enrolment in two Every Child Succeeds home visitation programs and interviewed at 9-12 months postpartum.  Depression assessed using the Beck Depression Inventory (14 item, cut-off of 13/14).  Lifetime and past year physical partner violence assessed using the Trauma Inventory. | 731 | With depression:  195/731 (26.7%)  Without depression:  536/731 (73.3%) | Lifetime:  207/731 (28.3%)  Past year violence:  117/731 (16.0%) | *Lifetime:*  *Physical*  With depression:  73/195 (37.4%) Without depression:  134/536 (25.0%)  OR: 1.8 (1.2-2.6)  P=0.001  *Past year:*  With depression:  43/195 (22.21%)  Without depression:  74/536 (13.8%)  OR: 1.8 (1.1-2.7)  P=0.007 | Total score:  29/42  Selection quality score:  8/14  Measurement quality score:  11/14 | |
| Beydoun 2010.[^43^](#_ENREF_84) | | Canada | Analysis of data from the Maternity Experiences Survey. Women sampled using the Canadian Census of Population and interviewed at home at 5-9 months postpartum.  Depression assessed using the Edinburgh Postnatal Depression Scale (10 item, cut-off 12/13).  Physical and sexual partner violence in the past 2 years assessed using Maternity Experiences Survey violence questions. | 6313 | With depression:  471/6313 (7.5%)  Without depression: 5842/6313 (92.5%) | Past 2 years:  357/6313 (5.7%) | *Past 2 years:*  With depression:  64/471(13.6%)  Without depression:  293/5842 (5.0%)  OR: 3.0 (2.2-4.0)  P<0.001 | Total score:  35/42  Selection quality score:  11/14  Measurement quality score:  12/14 | |
| Certain 2008.[^46^](#_ENREF_85) | | USA | Women recruited from 35 obstetric and gynaecology clinics and interviewed at an average of 6 weeks postpartum.  Depression assessed using the Edinburgh Postnatal Depression Scale (10 item, cut-off 12/13).  Past year physical and psychological domestic violence assessed using questions adapted from the Abuse Assessment Screen. | 1476 | With depression:  153/1476 (10.4%)  Without depression:  1323/1476 (89.6%) | Past year:  110/1476 (7.5%) | *Past year:*  With depression:  37/153 (24.2%)  Without depression:  73/1323 (5.5%)  OR: 5.5 (3.4-8.6)  P<0.001 | Total score:  31/42  Selection quality score: 8/14  Measurement quality score: 12/14 | |
| Cerulli 2011.[^47^](#_ENREF_47) | | USA | Women recruited from a university paediatric primary care clinic and interviewed <12 months postpartum.  Depression assessed using the Structured Clinical Interview for DSM-IV  Past year physical, sexual or psychological partner violence assessed using a previously piloted questionnaire. | 188 | With depression:  106/188 (56.4%)  With other disorder:  21/188 (11.2%)  Without disorder:  61/188 (32.4%) | Past year:  40/188 (21.3%) | *Past year:*  With depression:  31/106 (29.2%)  Without disorder:  8/61 (13.1%)  OR: 2.7 (1.1-7.4)  P=0.02 | Total score:  25/42  Selection quality score:  5/14  Measurement quality score:  9/14 | |
| Cwikel 2003.^49^ | | Israel | Bedouin women recruited from health centre. Women interviewed up to 12 months postpartum.  Depression assessed using the Centre for Epidemiologic Studies Depression Scale (6 item, cut-off 4/5)  Past year physical, sexual or psychological partner violence assessed with questions taken from the Commonwealth Fund survey. | 114 | With depression:  27/114 (23.7%)  Without depression:  87/114 (76.3%) | Past year:  Any violence:  37/114 (32.5%)  Physical:  19/114 (16.7%)  Sexual:  7/114 (6.1%)  Psychological:  31/114 (27.2%) | *Past year:*  *Any violence:*  With depression:  17/27 (63.0%)  Without depression:  20/87 (23.0%)  OR: 5.7 (2.1-16.1)  P<0.001  *Physical:*  With depression:  12/27 (44.4%)  Without depression:  7/87 (8.0%)  OR:9.1 (2.7-31.6)  P<0.001  *Sexual:*  With depression: 5/27 (18.5%)  Without depression: 2/87 (2.3%)  OR: 9.7 (1.4-105.0)  P=0.002  *Psychological:*  With depression:  17/27 (63.0%)  Without depression:  14/87 (16.1%)  OR: 8.9 (3.0-26.2)  P<0.001 | Total score:  18/42  Selection quality score:  6/14  Measurement quality score:  6/14 | |
| DeKlyen 2006.[^50^](#_ENREF_46) | | USA | Analysis of data from the Fragile Families and Child Wellbeing Study. Women recruited from hospital postnatal wards and interviewed at 12 months postpartum.  Depression assessed using the Composite International Diagnostic Interview-Short Form.  Lifetime physical violence by the child’s father assessed using the authors’ own questions. | 3077 | With depression:  439/3077 (14.3%)  With other disorder:  76/3077 (2.5%)  Without disorder: 2619/3077 (85.1%) | Lifetime:  Physical: 208/3077 (6.8%) | *Lifetime:*  With depression:  39/439 (8.9%)  Without disorder:  166/2619 (6.3%)  OR: 1.4 (1.0-2.1)  P=0.05 | Total score:  34/42  Selection quality score:  13/14  Measurement quality score:  10/14 | |
| Dennis (In press).[^51^](#_ENREF_86) | | Canada | Women recruited via mail-outs from family physicians, obstetricians and midwifes at >32 weeks gestation and interviewed at 8 weeks postpartum.  Depression assessed using the Edinburgh Postnatal Depression Scale (10 item, cut-off 12/13).  Lifetime physical and psychological partner violence assessed using the Antenatal Psychosocial Health Assessment. | 487 | With depression:  38/487 (7.8%)  Without depression:  449/487 (92.2%) | Lifetime:  Physical: 29/487 (6.0%)  Psychological: 54/487 (11.1%) | *Lifetime:*  *Physical:*  With depression:  5/38 (13.2%)  Without depression:  24/449 (5.3%)  OR: 2.7 (0.8-7.8)  P= 0.05  *Psychological:*  With depression:  9/37 (24.0%)  Without depression:  45/448 (10.0%)  OR: 2.9 (1.1-6.8)  P=0.008 | Total score:  31/42  Selection quality score:  10/14  Measurement quality score:  11/14 | |
| Gao 2010.[^56^](#_ENREF_87) | | New Zealand | Women recruited at a hospital birthing unit and interviewed at home approximately 6 weeks postpartum.  Depression assessed using the Edinburgh Postnatal Depression Scale (10 item, cut-off 12/13).  Past year physical and psychological partner violence assessed using the Conflict Tactics Scale. | 1085 | With depression:  165/1085 (15.2%)  Without depression:  920/1085 (84.8%) | Past year:  Physical: 250/1085 (23.0%)  Psychological: 836/1085 (77.1%) | *Past year:*  *Physical:*  With depression:  74/165 (44.8%)  Without depression:  176/920 (19.1%)  OR: 3.4 (2.4-4.9)  P<0.001  *Psychological:*  With depression:  139/165 (84.2%)  Without depression:  697/920 (75.8%)  OR: 1.7 (1.1-2.8)  P<0.001 | Total score:  29/42  Selection quality score:  10/14  Measurement quality score:  11/14 | |
| Gomez-Belos 2009.[^60^](#_ENREF_88) | | Peru | Women recruited on postnatal wards of a hospital specializing in high-risk pregnancies and interviewed <1 week postpartum.  Depression assessed using the Patient Health Questionnaire-9 (9 item, cut-off 14/15)  Lifetime partner violence and partner violence during pregnancy assessed using authors’ own questions. | 2317 | With depression:  330/2317 (14.2%)  Without depression  1987/2317 (85.8%) | Lifetime:  1031/2317 (44.5%)  During pregnancy:  486/2317 (21.0%) | *Lifetime:*  With depression:  220/330 (66.7%)  Without depression:  811/1987 (40.8%)  OR: 2.9 (2.3-3.7)  P<0.001  *During pregnancy:*  With depression:  131/330 (39.7%)  Without depression  355/1987 (17.9%)  OR: 3.0 (2.3-3.9)  P<0.001 | Total score:  31/42  Selection quality score:  6/14  Measurement quality score:  13/14 | |
| Ho-Yen 2007.[^64^](#_ENREF_90) | | Nepal | Women recruited from one urban hospital clinic, two rural health clinics, and during urban house-to house visits, and interviewed at 5-10 weeks postpartum.  Depression assessed using the Nepalese (validated) version of the Edinburgh Postnatal Depression Scale (10 item, cut-off 12/13).  Lifetime spousal violence assessed using authors’ own questions. | 425 | With depression: 21/425(4.9%)  Without depression:  404/425 (95.1%) | *Lifetime:*  30/425 (7.1%) | *Lifetime:*  With depression:  7/21 (33.3%)  Without depression:  23/404 (5.7%)  OR: 8.3 (2.6-24.4)  P<0.001 | Total score: 26/42  Selection quality score: 7/14  Measurement quality score: 11/14 | |
| Husain 2006.[^65^](#_ENREF_91) | | Pakistan | Women sampled from registers of Lady Health Workers and assessed at 3 months postpartum.  Depression assessed using the Edinburgh Postnatal Depression Scale (10 item, cut-off 11/12).  Past year domestic violence assessed using authors’ own questions. | 149 | With depression:  53/149 (35.6%)  Without depression:  96/149 (64.4%) | Past year:  3/149 (2.0%) | *Past year:*  With depression:  3/53 (5.7%)  Without depression:  0/96 (0.0%)  OR: n/a | Total score: 27/42  Selection quality score: 8/14  Measurement quality score: 10/14 | |
| Kornfeld 2012.^73^ | | USA | Analysis of records from universal screening of women attending an urban pediatric primary care clinic. Women screened at 2 to 6 months postpartum.  Depression assessed using the Whoolley questions (2 item).  Past year physical, sexual and psychological partner violence assessed using the 5-item Universal Violence Prevention Screening Protocol. | 173 | With depression:  43/173 (24.8%)  Without depression: 130/173 (75.1%) | Past year:  12/173 (6.9%) | With depression:  7/43 (16.3%) Without depression:  5/130 (3.8%)  OR: 4.9 (1.3-20.4)  P=0.005 | Total score:  23/42  Selection quality score:  5/14  Measurement quality score:  9/14 | |
| Lobato 2011.[^75^](#_ENREF_45) | | Brazil | Women recruited from public primary healthcare clinics serving predominantly low and moderate income clients and interviewed <5 months postpartum.  Depression assessed using the Portuguese (validated) version of the Edinburgh Postnatal Depression Scale (10 item, cut-off 11/12).  Physical and psychological partner violence during pregnancy assessed using the Conflict Tactics Scale-Revised. | 811 | With depression:  197/811 (24.3%)  Without depression:  614/811 (75.7%) | During pregnancy:  Physical: 307/811 (37.8%)  Psychological: 665/811 (82.0%) | *During pregnancy:*  *Physical:*  With depression:  121/197 (61.4%)  Without depression:  186/614 (30.3%)  OR: 3.7 (2.6-5.2)  P<0.001  *Psychological:*  With depression:  181/197 (97.9%)  Without depression:  484/614 (78.8%)  OR: 3.0 (1.7-5.6)  P<0.001 | Total score:  33/42  Selection quality score:  9/14  Measurement quality score:  14/14 | |
| McGarry 2009.[^80^](#_ENREF_92) | | USA | Analyses data from the Utah Pregnancy Risk Assessment and Monitoring System (PRAMS). Analysis restricted to women who reported postpartum depression but did not seek help for depression during pregnancy from a healthcare provider. Data are weighted for sample design and non-response.  Depression assessed using the Patient Health Questionnaire-2 (2 item, endorsement of “always” or “often” to either question).  Psychological partner violence during pregnancy assessed using authors’ (PRAMS) own questions. | 4433 | With depression  4433/4433 (100.0%)  Without depression:  0/4433 (0.0%) | During pregnancy:  836/4433 (18.8%) | *During pregnancy:*  With depression:  836/4443 (18.8%)  Without depression:  n/a  OR: n/a | Total score:  34/42  Selection quality score:  9/14  Measurement quality score:  11/14 | |
| Melo Jr. 2012^81^ | | Brazil | Women recruited in antenatal clinics and interviewed at 4-6 weeks postpartum.  Depression assessed using the Brazilian Edinburgh Postnatal Depression Scale (10 item, validated cut-off 11/12).  Lifetime physical, sexual and psychological partner violence assessed using questions from the WHO multi-country study on women’s health and domestic violence. | 555 | With depression:  60/555 (10.8%)  Without depression:  495/555 (89.2%) | Lifetime:  *Physical*  11/555 (2.0%)  *Sexual*  2/555 (0.4%)  *Psychological*  153/555 (27.6%) | *Lifetime:*  *Physical*  With depression:  Data not available  Without depression:  Data not available  OR: 4.5 (2.2-9.0)  P<0.05  *Sexual*  With depression:  Data not available  Without depression:  Data not available  OR: 4.7 (1.1-19.2)  P<0.05  *Psychological*  With depression:  Data not available  Without depression:  Data not available  OR: 3.9 (2.4-6.4)  P<0.05 | Total score:  25/42  Selection quality score:  5/14  Measurement quality score:  12/14 | |
| Pooler 2013^88^ | | USA | Analyses data of 22 US states included in the Pregnancy Risk Assessment and Monitoring System (PRAMS). Analysis restricted to women who whose participation/eligibility in the Special Supplemental Nutrition Program for Women, Infants and Children (WIC) could be verified; women whose infants were alive and residing with them at the time of the 2006-2008 survey. Data are weighted for sample design and non-response.  Depression assessed using the Patient Health Questionnaire-2 (2 item, endorsement of “always” or “often” to either question).  Physical partner violence during pregnancy assessed using authors’ (PRAMS) own questions. | 75,234 | With depression:  11001/75234 (13.8%)  Without depression:  64233/75234 (86.2%) | During pregnancy:  2668/75234 (3.4%) | *During pregnancy:*  With depression:  967/11001 (8.8%)  Without depression:  1701/64233 (2.6%)  OR: 3.6 (3.3-3.9)  P<0.001  AOR: 1.89 (1.59-2.24)  P<0.0001 | Total score:  34/42  Selection quality score:  13/14  Measurement quality score:  11/14 | |
| Quelopana 2012^89^ | | Chile | Women recruited from primary care clinics and interviewed at 2-6 weeks postpartum.  Depression assessed using the Spanish Postpartum Depression Screening Scale (35 item, validated cut-off 59/60).  Physical, sexual and psychological partner violence during the lifetime and during pregnancy assessed using the Women Abuse Screen. | 163 | With depression:  73/163 (44.8%)  Without depression:  90/163 (55.2%) | Lifetime:  105/163 (64.4%)  During pregnancy:  46/163 (28.2%) | *Lifetime*  With depression:  61/73 (83.6%)  Without depression: 44/90 (48.9%)  OR: 5.3 (2.4-12.2)  P<0.001  *During pregnancy*  With depression:  35/73 (47.9%)  Without depression:  11/90 (12.2%)  OR: 6.6 (2.9-15.9)  P<0.001 | Total score:  32/42  Selection quality score:  6/14  Measurement quality score:  14/14 | |
| Tiwari 2008.[^98^](#_ENREF_93) | | Hong Kong | Women recruited through hospital obstetrics and gynaecology departments and interviewed at home at 1 week postpartum.  Depression assessed using the Edinburgh Postnatal Depression Scale (Chinese) (10 item, cut-off 9/10) .  Past year physical, sexual and psychological partner violence assessed using the Abuse Assessment Screen (Chinese). | 3245 | With depression:  2122/3245 (65.4%)  Without depression:  1123/3245 (34.6%) | Past year:  *Physical/sexual:* 80/3245 (2.5%)  *Psychological only:*  216/3245 (6.7%) | *Past year:*  *Physical/sexual:*  With depression: not available  Without depression: not available  OR: 1.7 (0.8-3.7)  P=0.14  *Psychological*  With depression: not available  Without depression: not available  OR: 1.8 (1.1-3.0)  P=0.02 | Total score:  30/42  Selection quality score:  7/14  Measurement quality score:  11/14 | |
| Woolhouse 2012.[^100^](#_ENREF_50) | | Australia | Women sampled from public hospitals’ registration data and mailed self-completion questionnaires at birth and completed questionnaires at <24 weeks gestation and 3, 6, and 12 months postpartum.  Depression assessed at 12 months using the Edinburgh Postnatal Depression Scale (10 item, cut-off 12/13).  Past year physical and psychological partner violence assessed at 12 months using the Composite Abuse Scale. | 1303 | With depression:  102/1303 (7.8%)  Without depression:  1201/1303 (92.2%) | Past year: 216/1303 (7.8%) | *Past year:*  With depression:  41/102 (40.2%)  Without depression:  175/1201 (14.6%)  OR: 3.9 (2.5-6.2)  P<0.001 | Total score:  35/42  Selection quality score:  10/14  Measurement quality score:  14/14 | |
| **Postnatal anxiety** | | | | | | | | | |
| DeKlyen 2006.[^50^](#_ENREF_46) | |  | Analysis of data from the Fragile Families and Child Wellbeing Study. Women recruited from hospital postnatal wards and interviewed at 12 months postpartum.  Anxiety assessed using the Composite International Diagnostic Interview-Short Form.  Lifetime physical violence by the child’s father assessed using the authors’ own questions. | 3077 | With anxiety:  76/3077 (2.5%)  With other disorder:  382/3077 (12.4%)  Without disorder:  2619/3077 (85.1%) | Lifetime: 208/3077 (6.8%) | *Lifetime:*  With anxiety:  21/76 (27.6%)  Without disorder:  166/2619 (6.3%)  OR: 5.6 (3.2-9.7)  P<0.001 | Total score:  34/42  Selection quality score:  13/14  Measurement quality score:  10/14 | |
| **Postnatal post-traumatic stress disorder** | | | | | | | | | |
| Cerulli 2011.[^47^](#_ENREF_47) | | USA | Women recruited from a university paediatric primary care clinic and interviewed <12 months postpartum.  PTSD assessed using the Structured Clinical Interview for DSM-IV  Past year partner violence assessed using a previously piloted questionnaire. | 188 | With PTSD:  17/188 (9.0%)  With other disorder:  110/188 (9.3%)  Without disorder:  61/188 (32.4%) | Past year: 40/188 | *Past year:*  With PTSD:  7/17 (41.2%)  Without disorder:  8/61 (13.1%)  OR: 4.6 (1.1-18.4)  P=0.009 | Total score:  25/42  Selection quality score:  5/14  Measurement quality score:  9/14 | |
| **Postnatal psychological distress** | | | | | | | | | |
| Ali 2009.[^40^](#_ENREF_94) | | Pakistan | Women recruited during house to house visits and interviewed at 1, 2, 6, and 12 months postpartum.  Psychological distress assessed using the Aga Khan University Anxiety and Depression Scale (cut-off 19/20).  Lifetime physical or psychological abuse by a family member assessed using authors’ own questions. | 267 | With psychological distress at any interview:  77/267 (28.8%)  Without psychological distress at any interview:  190/267 (71.2%) | Lifetime: 26/267 (9.7%) | *Lifetime:*  With distress:  13/77 (16.9%)  Without distress:  13/190 (6.8%)  OR: 2.8 (1.1-6.8)  P=0.01 | Total score: 29/42  Selection quality score: 8/14  Measurement quality score: 9/14 | |
| Pollock 2009.[^87^](#_ENREF_95) | | Mongolia | Women recruited in hospital and interviewed at home approximately 8 weeks postpartum.  Psychological distress assessed using the Self-Reporting Questionnaire-20 (20 item, no information on cut-off).  Lifetime physical abuse by family members assessed using author’s own questions. | 1035 | With psychological distress:  92/1035 (8.9%)  Without psychological distress:  943/1035 (91.1%) | Lifetime: 35/1035 (3.4%) | *Lifetime:*  With distress:  12/92 (13.0%)  Without distress:  23/943 (2.4%)  OR: 6.0 (2.6-13.1)  P<0.001 | Total score:  27/42  Selection quality score:  9/14  Measurement quality score:  9/14 | |
| Romito 2009.[^93^](#_ENREF_96) | | Italy | Women recruited in hospital postnatal wards. Interviews conducted at 8 months postpartum.  Psychological distress assessed using the General Health Questionnaire-12 (12 item, cut-off of 5/6).  Past year partner and family physical, sexual and psychological violence assessed using authors own questions. | 290 | Psychological distress:  15/290 (5.2%)  No psychological distress:  275/290 (94.8%) | Past year (partner):  15/290 (5.2%)  Past year (family):  17/290 (5.9%) | *Past year (partner):*  With distress:  4/15 (26.7%)  Without distress:  11/275 (4.0%)  OR: 8.7 (1.7-35.7)  P=0.001  *Past year (family):*  With distress:  5/15 (33.3%)  Without distress:  12/275 (4.4%)  OR: 11.0 (2.5-41.9)  P<0.001 | Total score:  28/42  Selection quality score:  6/14  Measurement quality score:  8/14 | |
| Saurel-Cubizolles 1997.[^94^](#_ENREF_97) | | France | Women recruited from 3 public maternity hospitals and interviewed at 12 months postpartum.  Psychological distress assessed using the General Health Questionnaire-12 (12 item).  Past year marital violence assessed using authors’ own questions. | 621 | Psychological distress: 66/621(10.6%)  No psychological distress: 555/621 (8.9%) | Past year: 17/621 (2.7%) | *Past year:*  With distress:  8/66 (12.1%)  Without distress:  9/555 (1.6%)  OR: 8.4 (2.7-25.3)  P<0.001 | Total score:  28/42  Selection quality score:  7/14  Measurement quality score:  8/14 | |
| **Perinatal mental disorder** | | | | | | | | | |
| Fisher 2010.^54^ | | Vietnam | Women recruited from community health centres and interviewed at >30 weeks gestation or between 4-8 weeks postpartum.  Depression assessed using Structured Clinical Interview for DSM-IV.  Past year marital physical and psychological violence assessed using authors’ own questions. | 364 | With depression:  53/364 (14.6%)  With other disorders: 56/364 (15.4%)  Without disorder: 255/364 (70.1%) | Past year:  *Any violence:*  56/364 (15.4%)  *Physical:*  10/364 (2.7%)  *Psychological*  50/364 (13.7%) | *Past year:*  *Any violence:*  With depression:  n=21/53 (39.6%)  Without disorder:  n=35/255 (13.7%)  OR: 4.1 (2.0 – 8.3)  P<0.001  *Physical:*  With depression:  n=5/53 (9.4%)  Without disorder  n=5/255 (2.0%)  OR: 5.2 (1.1 – 23.4)  P=0.005  *Psychological:*  With depression:  n=18/53 (34.0%)  Without depression:  n=32/255 (12.5%)  OR: 3.6 (1.7 – 7.4)  P<0.001 | Total score:  33/42  Selection bias:  12/14  Measurement bias:  11/14 | |
| Fisher 2010.[^54^](#_ENREF_98) | | Vietnam | Women recruited from community health centres and interviewed at >30 weeks gestation or between 4-8 weeks postpartum.  Anxiety assessed using Structured Clinical Interview for DSM-IV.  Past year marital physical and psychological violence assessed using authors’ own questions. | 364 | Generalised Anxiety Disorder (GAD)  With anxiety:  10/364 (2.7%)  With other disorders: 99/364 (27.2%)  Without disorder: 255/364 (70.1%) | Past year:  *Any violence:*  56/364 (15.4%)  *Physical:*  10/364 (2.7%)  *Psychological:*  50/364 (13.7%) | *Past year:*  *Any violence:*  With anxiety:  2/10 (20.0%)  Without disorder:  35/255 (13.7%)  OR: 1.6 (0.2 – 8.3)  P=0.57  *Physical:*  With anxiety:  0/10 (0.0%)  Without disorder:  5/255 (2.0%)  OR: n/a  *Psychological:*  With anxiety:  2/10 (20.0%)  Without disorder:  32/255 (12.5%)  OR: 1.7 (0.2 – 9.3)  P=0.49 | Total score:  33/42  Selection bias:  12/14  Measurement bias:  11/14 | |

*type of violence provided where available in original studies
