# Supplementary material for: Domestic Violence and Perinatal Mental Disorders: A Systematic Review and Meta-Analysis
Source: PLoS Med. 2013 May 28;10(5):e1001452. doi: 10.1371/journal.pmed.1001452 (PMC3665851; doi:10.1371/journal.pmed.1001452)
Supplement: Table S2 — Characteristics and reported outcomes of longitudinal analyses of included studies. (DOCX) [file pmed.1001452.s003.docx]

**Table S2: Characteristics and reported outcomes of longitudinal analyses from included studies**

| **Author and year** | **Country** | **Method** | **Sample size** | **Frequency of disorder** | **Type of domestic violence*** | **Prevalence and odds of violence** | **Quality appraisal scores** |
| --- | --- | --- | --- | --- | --- | --- | --- |
| **Antenatal violence as a risk factor for depression** | | | | | | | |
| Budhathoki  2012.^45^ | Nepal | Women recruited from antenatal clinics.  1^st^ interview conducted during the third trimester. Physical, sexual and psychological marital violence assessed using questions from the WHO multi-country study on women’s health and domestic violence.  2^nd^ interview conducted at 6 weeks postpartum. Depression assessed using Nepalese Edinburgh Postnatal Depression Scale (10 item, cut-off 12/13).  3^rd^ interview conducted at 10 weeks postpartum. Depression assessed using Nepalese Edinburgh Postnatal Depression Scale (10 item, cut-off 12/13). | 72 | *6 weeks*  With depression:  14/72 (19.4%)  Without depression:  58/72 (80.6%)  *10 weeks*  With depression:  16/72 (22.2%)  Without depression:  56/72 (77.8%) | *Lifetime*  Physical  15/72 (20.8%)  Sexual  10/72 (13.4%)  Psychological  14/72 (19.4%) | *Lifetime*  *6 weeks*  Physical  With depression:  4/14 (28.6%)  Without depression:  11/58 (19.0%)  OR: 1.7 (0.3-7.4)  P=0.33  Sexual  With depression:  2/14 (14.3%)  Without depression:  8/58 (13.8%)  OR: 1.0 (0.1-6.2)  P=0.63  Psychological  With depression:  3/14 (21.4%)  Without depression:  11/58 (19.0%)  OR:1.2 (0.2-5.5)  P=0.55  *10 weeks*  Physical  With depression:  4/16 (25.0%)  Without depression:  11/56 (19.6%)  OR: 1.4 (0.3-5.7)  P=0.44  Sexual  With depression:  1/16 (6.3%)  Without depression:  9/56 (16.1%)  OR: 0.3 (0.0-2.9)  P=0.29  Psychological  With depression:  4/16  Without depression:  10/56  OR: 1.5 (0.3-6.6)  P=0.38 | Total score:  22/42  Selection quality score:  6/14  Measurement quality score:  10/14 |
| Dolatian 2010.[^102^](#_ENREF_99) | Iran | Women recruited from health centres.  1^st^ interview conducted in the last month of gestation. Marital violence assessed using a combination of questions from the Abuse Assessment Screen, Abusive Behaviour Inventory, Composite Abuse Scale, Measure of Wife Abuse, Revised Conflict Tactics Scale and Severity of Violence Against Women Scale.  2^nd^ interview conducted at 4-6 weeks postpartum. Depression assessed using the Edinburgh Depression Scale (10 item, cut-off 9/10). | 240 | With depression:  82/240 (34.2%)  Without depression:  158/240 (65.8%) | Lifetime:  120/240 (50.0%) | *Lifetime:*  With depression:  63/82 (76.8%)  Without depression:  57/158 (36.1%)  OR: 5.9 (3.1-11.4)  P<0.001 | Total score:  22/42  Selection quality score:  5/14  Measurement quality score:  9/14 |
| Fisher 2012.^55^ | Vietnam | Women recruited by healthcare workers during routine household visits.  1^st^ interview conducted at 12-20 weeks gestation. Lifetime physical, sexual, and psychological partner violence assessed using authors’ own questions.  3^rd^ and 4^th^ interviews conducted at 2 months and 6 months postpartum. Depression assessed using Vietnamese Edinburgh Postnatal Depression Scale (10 item, cut-off 3/4).  Odds ratios adjusted for woman’s age, education level and occupation, parity, history of miscarriage or stillbirth, household wealth index, quality of relationship with mother and mother-in-law, and welcome pregnancy. | 419 | With depression:  Without depression: | *Lifetime:*  Physical:  79/417 (19.0%)  Sexual:  28/417 (6.7%)  Psychological:  63/417 (15.2%) | *Lifetime*  *Physical*  With depression:  Data not available  Without depression:  Data not available  AOR: 2.6 (1.5-4.5)  P<0.05  *Sexual*  With depression:  Data not available  Without depression:  Data not available  AOR: 3.9 (1.8-8.7)  P<0.05  *Psychological*  With depression:  Data not available  Without depression:  Data not available  AOR: 3.1 (1.8-5.8)  P<0.05 | Total score:  31/42  Selection quality score:  9/14  Measurement quality score:  12/14 |
| Flach 2011.[^19^](#_ENREF_21) | UK | Women recruited during pregnancy as part of the Avon Longitudinal Study of Parents and Children.  1^st^ interview conducted at 18 weeks gestation. Physical and psychological partner violence during pregnancy assessed using authors’ own questions.  Follow up interviews conducted at 8 weeks postpartum. Depression assessed using the Edinburgh Postnatal Depression Scale (10 item, cut-off 12/13). | 13617 | With depression:  1504/13617 (11.0%)  Without depression:  12113/13617 (89.0%) | During pregnancy:  953/13617 (7.0%) | *During pregnancy:*  *Physical and psychological:*  With depression:  238/1504 (15.8%)  Without depression:  715/12113 (5.9%)  OR: 3.0 (2.6-3.5)  P<0.001 | Total score:  35/42  Selection quality score:  13/14  Measurement quality score:  12/14 |
| Gausia 2009.[^58^](#_ENREF_100) | Bangladesh | Women sampled from primary healthcare clinic records and recruited and interviewed at home.  1^st^ interview at 34-35 weeks gestation. Lifetime physical marital violence assessed using authors’ own questions.  2^nd^ interview conducted at 6-8 weeks postpartum. Depression assessed using the Bangla Edinburgh Postnatal Depression Scale (10 item, validated cut-off 9/10). | 346 | With depression:  76/346 (22.0%)  Without depression:  270/346 (78.0%) | Lifetime:  16/346 (4.6%) | *Lifetime:*  With depression:  8/76 (10.5%)  Without depression:  8/270 (3.0%)  OR:3.9 (1.2-12.2)  P=0.006 | Total score:  31/42  Selection quality score:  10/14  Measurement quality score:  11/14 |
| Leung 2002.[^74^](#_ENREF_44) | China | Women recruited from hospital postnatal ward.  1^st^ interview conducted at 2 days postpartum. Past year partner and family violence assessed using a modified version of the Abuse Assessment Screen.  Follow-up interviews conducted at 6 weeks postpartum. Depression assessed using the Chinese (validated) version of the Edinburgh Postnatal Depression Scale (10 item, cut-off 9/10). | 694 | With depression:  50/694 (7.2%)  Without depression:  644/694 (92.3%) | Past year: 113/694 (16.3%) | *Past year:*  With depression:  17/50 (34.0%)  Without depression:  96/644 (14.9%)  OR: 2.9 (1.5-5.7)  P<0.001 | Total score:  28/42  Selection quality score:  9/14  Measurement quality score:  9/14 |
| Ludermir 2010.[^76^](#_ENREF_101) | Brazil | Women recruited from Primary Healthcare Programme records.  1^st^ interview conducted in third trimester. Physical, sexual and psychological partner violence during pregnancy assessed using the WHO Multi Country Study on Women’s Health and Domestic Violence questionnaire.  2^nd^ interview conducted 3-6 months postpartum. Depression assessed using the Portuguese version of the Edinburgh Postnatal Depression Scale (10 item, validated cut-off 11/12). | 1045 | With depression:  270/1045 (25.8%)  Without depression:  775/1045 (74.2%) | During pregnancy:  321/1045 (30.7%) | *During pregnancy:*  With depression:  139/270 (51.5%)  Without depression:  182/775 (23.5%)  OR: 3.5 (2.6-4.7)  P<0.001 | Total score:  36/42  Selection quality score:  11/14  Measurement quality score:  13/14 |
| Malta 2012.^77^ | Canada | Women recruited from healthcare offices, community posters, health services website and laboratory services.  2^nd^ interview conducted at 34-36 weeks gestation. Lifetime physical, sexual, psychological, and financial abuse or neglect by partner assessed using authors’ own questions.  3^rd^ interview conducted at 4 months postpartum. Depression assessed using the Edinburgh Postnatal Depression Scale (10 item, cut-off 12/13). Anxiety assessed using the Spielberger State Anxiety Index (cut-off 39/40). | 1382 | With depression:  71/1382 (5.1%)  Without depression or anxiety:  1112/1382 (80.5%) | Lifetime:  110/1183 (9.3%) | With depression:  13/71 (18.3%)  Without depression or anxiety:  97/1112 (8.7%)  OR: 2.3 (1.1-4.5)  P=0.007 | Total score:  28/42  Selection quality score:  4/14  Measurement quality score:  13/14 |
| Manzolli 2012^78^ | Brazil | Women recruited from antenatal clinics.  1^st^ interview conducted at 16 to 36 weeks gestation. Physical, sexual and psychological partner during pregnancy and lifetime assessed using the Abuse Assessment Screen.  3^rd^ interview conducted at 4^th^ month postpartum. Depression assessed using the Brazilian Primary Care Evaluation of Mental Disorders depression module (9 items, cut-off 5/6). | 375 | With depression:  111/375 (29.6%)  Without depression:  264/375 (70.4%) | Lifetime:  174/375 (46.7%)  During pregnancy:  71/375 (18.9%) | *Lifetime*  With depression:  74/111 (66.7%) Without depression:  100/264 (37.9%)  OR: 3.3 (2.0-5.4)  P<0.001  *During pregnancy:*  With depression:  34/111 (30.6%) Without depression:  37/264 (14.0%)  OR: 2.7 (1.5-4.8)  P<0.001 | Total score:  27/42  Selection quality score:  5/14  Measurement quality score:  12/14 |
| Patel 2002.[^86^](#_ENREF_54) | India | 1^st^ interview conducted at >30 weeks gestation. Marital violence (lifetime and during pregnancy) assessed using author’s own questions.  2^nd^ interview conducted at 6-8 weeks postpartum. Depression assessed using the Edinburgh Postnatal Depression Scale – Konkani Version (10 item, validated cut-off 11/12).  3^rd^ interview conducted at 6 months postpartum. Depression assessed using the Edinburgh Postnatal Depression Scale – Konkani Version (10 item, validated cut-off 11/12). | 252 | *6-8 weeks:*  With depression:  59/252 (23.4%)  Without depression:  193/252 (76.6%)  *6 months:*  With depression:  51/235 (21.7%)  Without depression:  184/235 (78.3%) | Lifetime:  31/235 (13.2%)  During pregnancy:  15/235 (6.4%) | *6-8 weeks:*  *Lifetime:*  With depression:  14/58 (24.1%)  Without depression:  17/174 (9.8%)  OR: 2.9 (1.2-6.9)  P=0.005  *Pregnancy:*  With depression:  9/58 (15.5%)  Without depression:  6/174 (3.5%)  OR: 5.1 (1.5-18.3)  P=0.001  *6 months:*  *Lifetime*  With depression:  17/51 (33.3%)  Without depression:  14/184 (7.6%)  OR: 6.1 (2.5-14.6)  P<0.001  *Pregnancy:*  With depression:  10/51 (19.6%)  Without depression:  5/184 (2.7%)  OR: 8.7 (2.5-33.9)  P<0.001 | Total quality:  23/42  Selection quality:5/14  Measurement quality: 10/14 |
| Rodriguez 2010.^92^ | USA | Women recruited from antenatal clinics at 2 private healthcare organizations.  1^st^ interview conducted at >12 weeks gestation. Lifetime partner violence assessed using the Abuse Assessment Screen.  2^nd^ interview conducted at 3 and 7 months postpartum. Depression assessed using the Beck Depression Inventory Fast Screen (7 item, cut-off 3/4). | 210 | *3 months:*  With depression:  35/210 (16.6%)  Without depression:  175/210 (83.3%)  *7 months:*  With depression:  54/210 (25.7%)  Without depression:  156/210 (74.3%) | Lifetime:  92/210 (76.7%) | *3 months:*  With depression:  26/35 (74.3%)  Without depression:  66/175 (37.7%)  OR: 4.8 (2.0-12. 2)  P<0.001  *7 months:*  With depression:  39/54 (72.2%)  Without depression:  53/156 (34.0%)  OR: 5.1 (2.4-10.7)  P<0.001 | Total score:  31/42  Selection quality score:  8/14  Measurement quality score:  11/14 |
| Zhang 2011.^101^ | China | Women recruited from maternity wards.  1^st^ interview conducted pre-natally, after hospitalisation. Partner violence during current pregnancy or the preceding year assessed using the Abuse Assessment Screen.  2^nd^ interview conducted 4-6 weeks postpartum. Depression assessed using the Chinese Edinburgh Postnatal Depression Scale (10 item, validated cut-off 12/13). | 215 | With depression:  67/215(31.2%)  Without depression:  148/215 (68.8%) | Past 21 months:  81/215 (37.7%) | With depression:  54/67 (80.5%)  Without depression:  27/148 (18.2%)  OR: 17.1 (8.2-35.3)  P<0.001 | Total score:  32/42  Selection quality score:  6/14  Measurement quality score:  14/14 |
| **Antenatal anxiety as a risk factor for violence** | | | | | | | |
| Malta 2012.^77^ | Canada | Women recruited from healthcare offices, community posters, health services website and laboratory services.  2^nd^ interview conducted at 34-36 weeks gestation. Lifetime physical, sexual, psychological, and financial abuse or neglect by partner assessed using authors’ own questions.  3^rd^ interview conducted at 4 months postpartum. Anxiety assessed using the Spielberger State Anxiety Index (cut-off 39/40). Depression assessed using the Edinburgh Postnatal Depression Scale (10 item, cut-off 9/10). | 1382 | With anxiety:  206/1382 (14.9%)  Without depression or anxiety:  1112/1382 (80.5%) | Lifetime:  126/1318 (9.6%) | With depression:  29/126 (23.0%)  Without depression or anxiety:  97/1112 (8.7%)  OR: 1.7 (1.1-2.7)  P=0.02 | Total score:  28/42  Selection quality score:  4/14  Measurement quality score:  13/14 |
| **Antenatal depression as a risk factor for violence** | | | | | | | |
| Dunn  2004.[^52^](#_ENREF_48) | USA | Women recruited from prenatal clinics.  1^st^ interview conducted at <14 weeks gestation. Depression assessed using the Centre for Epidemiologic Studies Depression Scale (10 item, no information on cut-off).  2^nd^ interview conducted at >28 weeks gestation. Physical partner violence during pregnancy assessed using the Abuse Assessment Screen. | 439 | With depression:  154/439 (35.1%)  Without depression:  285/439 (64.9%) | During pregnancy:  29/439 (6.6%) | *During pregnancy:*  With depression:  Not available.  Without depression:  Not available.  OR:2.9 (1.9-4.3)  P<0.001 | Total score:  30/42  Selection quality score:  8/14  Measurement quality score:  13/14 |
| Manzolli 2012.^78^ | Brazil | Women recruited from antenatal clinics.  1^st^ interview conducted at 16 to 36 weeks gestation. Depression assessed using the Brazilian Primary Care Evaluation of Mental Disorders depression module (9 items, cut-off 5/6).  3^rd^ interview conducted at 4^th^ month postpartum. Physical, sexual and psychological partner during postpartum assessed using the Abuse Assessment Screen. | 375 | With depression:  143/375 (38.1%)  Without depression:  232/375 (61.9%) | Past 4 months:  83/375 (23.5%) | With depression:  45/143 (31.5%)  Without depression:  38/232 (16.4%)  OR: 2.3 (1.4-4.0)  P<0.001 | Total score:  27/42  Selection quality score:  5/14  Measurement quality score:  12/14 |
| Radestad 2004.[^90^](#_ENREF_49) | Sweden | Women recruited from antenatal clinics.  1^st^ interview conducted at first antenatal visit. Depression assessed using the Edinburgh Postnatal Depression Scale (10 item, cut-off of 14/15).  2^nd^ interview conducted at 12 months postpartum. Past year physical partner violence assessed using authors’ own questions. | 2427 | With depression:  160/2427 (6.6%)  Without depression:  2267/2427 (93.4%) | Past year:  52/2427 (2.1%) | *Past year:*  With depression:  13/160 (8.1%)  Without depression:  36/2267 (1.6%)  OR: 5.5 (2.6-10.9)  P<0.001 | Total score:  31/42  Selection quality score:  8/14  Measurement quality score:  11/14 |
| Records 2009.[^91^](#_ENREF_77) | USA | Women recruited from antenatal clinics.  1^st^ interview conducted at 3^rd^ trimester. Depression assessed using the Centre for Epidemiologic Studies Depression Scale (10 item, cut-off 15/16).  4^th^ interview conducted at 6 months postpartum. Psychological partner violence in the past two months assessed using the Severity of Violence Against Women Scale (cut-off 5/6). | 139 | With depression:  104/139 (74.8%)  Without depression:  35/139 (25.2%) | 2 months:  1/139 (0.7%) | *2 months:*  With depression:  1/104 (0.96%)  Without depression:  0/35 (0.0%)  OR: n/a | Total score:  30/42  Selection quality score:  7/14  Measurement quality score:  13/14 |
| Woolhouse 2012.[^100^](#_ENREF_50) | Australia | Women recruited by mail-out from public hospitals where they were registered to give birth.  1^st^ interview at <24 weeks gestation.  Depression assessed using the Edinburgh Postnatal Depression Scale (10 item, cut-off 12/13).  Follow up interviews conducted at 12 months postpartum. Past year physical and psychological partner violence assessed using the Composite Abuse Scale. | 1300 | With depression:  92/1300 (7.1%)  Without depression:  1208/1300 (93.0%) | Past year:  216/1300 (16.6%) | *Past year:*  With depression:  34/92 (37.0%)  Without depression:  182/1208 (15.1%)  OR: 3.3 (2.0-5.3)  P<0.001 | Total score:  35/42  Selection quality score:  10/14  Measurement quality score:  14/14 |

*type of violence provided where available in original studies
