# Supplementary material for: Domestic Violence and Perinatal Mental Disorders: A Systematic Review and Meta-Analysis
Source: PLoS Med. 2013 May 28;10(5):e1001452. doi: 10.1371/journal.pmed.1001452 (PMC3665851; doi:10.1371/journal.pmed.1001452)
Supplement: Text S2 — Systematic review protocol. (DOC) [file pmed.1001452.s005.doc]

**Text S2: Systematic Review Protocol**

1. **Title**

Systematic review of the prevalence and risk of domestic violence victimisation amongst people with mental disorders

1. **Review Team**

Principal Investigator: Professor Louise M. Howard

Reviewers: Siân Oram, Kylee Trevillion

1. **Background**

Domestic violence is a major public health problem, which affects the lives of hundreds of thousands of men and women each year. Victims of domestic violence have increased use of healthcare services compared to those not experiencing abuse: in the UK alone, the healthcare costs of physical injury are estimated at £1.2 billion per annum and an additional £176 million for mental healthcare costs (Walby, 2004). Often having serious and long term health consequences, domestic violence has been linked with many mental health problems including anxiety, depression, post-traumatic stress disorder, substance misuse and suicidal tendencies (Campbell, 2002; Golding, 1999). Research over the life-course has shown that prolonged exposure to threatening life events, including domestic violence, is associated with the chronicity (duration and recurrence) of mental disorders (Brown et al., 1994).

Recent reviews have suggested that experiences of domestic violence are more frequent among people with mental disorders (Friedman & Loue, 2007; Howard et al., 2010). However, these reviews did not critically appraise the methodological quality of primary studies or provide summary statistics using meta-analytic techniques. In addition, most reviews have focused on victimisation against women only (e.g. Golding 1999; Friedman and Loue 2007), despite the increased risk of victimisation among men with severe mental illness (Goodman et al., 2001; Teplin et al., 2005). Furthermore, limited consideration has been given to the substantial body of research on violent victimisation against people with mental disorders, from which data on the prevalence and risk of domestic violence victimisation could potentially be extracted.

Therefore, the aim of this review is to extend previous findings by systematically reviewing the evidence on the prevalence and risk of adulthood domestic violence victimisation experienced by people with mental disorders. A key component of this review will be to re-examine and update papers from recent high quality systematic reviews of the psychiatric literature measuring prevalence of violence perpetrated against people with mental disorders, to extract data on domestic violence available from these studies. A critical conceptual analysis, examining how domestic violence is measured and conceptualised (i.e. type and severity of violence) across studies and the potential effect this may have on reported outcomes will also be considered.

If possible (i.e. if primary studies are not too heterogeneous, too few in number or of too poor quality) a meta-analysis will be carried out to estimate the prevalence and risk of domestic violence victimisation among men and women with mental disorders.

**4. Objectives**

The objectives of this review are to establish:

1. Prevalence (adult lifetime and past year) of domestic violence victimisation amongst people with specific mental disorders
2. The relative risk of domestic violence victimisation amongst people with specific mental disorders compared with controls

Analyses of the prevalence and risk of domestic violence victimisation amongst people with mental disorders will be conducted separately according to gender, type and chronicity of mental disorder.

The key terms of the review are defined as follows:

**Domestic Violence**

The definition for domestic violence is that laid down by the Home Office, i.e., “any incident of threatening behaviour, violence or abuse (psychological, physical, sexual, financial or emotional) between adults who are or have been intimate partners or family members regardless of gender or sexuality” (HomeOffice 2005, p7)

The above definition has been chosen in preference to those produced by the United Nations and the World Health Organisation, which only consider violence perpetrated against women and would therefore exclude the experiences of men with mental disorders (United_Nations_General_Assembly 1993; WHO 1996). This definition, which is also used by the British Medical Association (BMA 2007), is broader than that adopted by the American Medical Association (AMA), which defines domestic violence in relation to intimate partner violence (AMA 1992). Therefore the UK Home Office definition is more applicable to this review, which examines abuse perpetrated by family members and carers, as well as by intimate partners, on both men and women.

**Mental disorder**

Mental disorder, for the purpose of this review, is defined as including the following diagnoses in accordance with ICD-10 or DSM-IV criteria (or earlier versions of the ICD and DSM classifications) (ICD-10 codes provided below):

*Schizophrenia, schizotypal and delusional disorders* (Schizophrenia [F20-21, 295.0-6, 295.8-9], other psychoses (schizoaffective disorder [F25, 295.7], paranoid states [297], other nonorganic psychoses [F28-F29, 298], persistent and induced delusional disorders [F22, F24], acute and transient psychotic disorders [F23]); *Mood [affective] disorders* (manic episode [F30, 296.00-.06], bipolar affective disorder [F31, 296.40-.89], depressive disorders [F32-33, 296.20-296.36, 311] (depression in pregnancy and postpartum depression will also be investigated separately), persistent mood [affective] disorder [F34, 300.04, 301.13], other mood [affective] disorder [F38-39]); *Neurotic, stress-related and somatoform disorders* (anxiety disorders [F40-42, 300.01-.03, 300.21-300.29], acute stress reaction [F43, 308.3], post-traumatic stress disorder [F43.1, 309.81], adjustment disorders [F43.2, 309.0-.4, 309.9], dissociative disorders [F44, 300.12-.15, 300.6], somatoform disorders [F45, 300.11, 300.7-300.81, 307.8] other neurotic disorders [F48]); *Eating disorders* [F50.0-F50.9]; and *personality disorders* [F60-61, 301.0-301.9].

N.B. This list excludes primary diagnoses of mental disorder due to psychoactive substance use.  Studies reporting on co-morbid substance use, however, are eligible for inclusion.

**5. Selection Criteria**

**5.1 Inclusion criteria**

Study Population

Studies will be eligible for inclusion in the review if samples include men and/or women who are 16 years or older and who have a mental disorder. If studies include people with mental disorders as a subset of a broader sample, the data on the specific disorders must be reported separately. Mental disorder must be assessed using a validated diagnostic or screening instrument.

Study Setting

No restrictions have been placed on study setting.

Study Characteristics

Studies are eligible for inclusion if they present the results of peer-reviewed research based on the following study designs: experimental studies (e.g. randomised controlled trials, non randomised controlled trials, parallel group studies), which measure prevalence of domestic violence as sub-analyses, before and after studies, interrupted time series studies, cohort studies, case-control studies, and cross-sectional studies.

Study Outcomes

To be eligible for inclusion in the review, studies must measure the prevalence of adult lifetime and/or past year domestic violence victimisation, and/or the risk of domestic violence victimisation (i.e., odds ratios, relative risk, attributable risk), or have collected data from which these statistics can be calculated.

**5.2 Exclusion criteria**

Study Population

Studies in which mental disorders have not been assessed using a validated screening or diagnostic instrument (i.e., which use self-report measures, case note review without use of a validated instrument such as OPCRIT (McGuffin et al., 1991) or reported clinical diagnosis without a validated instrument) will be excluded from the review. Studies will also be excluded if they include participants who are aged 15 years or younger and do not provide appropriate age-disaggregated data.

Study Characteristics

Studies using any of the following research designs will be excluded: case studies, case series, or any qualitative design (e.g., in depth interviews, focus groups). Theses/dissertations and published reports will be included. Single case studies, general discussion papers and comments or letters will be excluded, as will book chapters and conference papers.

Study Outcomes

Studies which do not measure the prevalence (adult lifetime or past year) or the risk (i.e., odds ratios, relative risk, attributable) of adult domestic violence victimisation amongst people with mental disorders (as defined in section 4) will be excluded from the review.

Studies that include participants with mental disorders (as defined in section 4) as part of a broader sample and do not present disaggregated measures of the prevalence or risk of domestic violence victimisation for specific diagnostic groups will be excluded, unless corresponding authors can provide the required information.

**5.3 Date Restrictions**

This review will examine all studies published up to the end of March 2011. No lower date restrictions will be assigned.

**5.4 Language Restrictions**

No language restrictions have been placed on this review.

**6. Search Strategy**

The review will attempt to locate all peer-reviewed published studies which meet the above inclusion criteria.

**6.1 Search Terms**

The search terms for domestic violence are adapted from published Cochrane protocols and peer-reviewed reviews (Dalsbo & Johme, 2006; Friedman & Loue, 2007; Ramsay et al., 2002). Search terms for mental disorders are adapted from NICE guidelines (NICE, 2008) (see appendix A).

**6.2 Data Sources**

The review will search multiple electronic databases and relevant websites. Reference lists of all included studies will also be searched and forward citation tracking used to identify additional potentially relevant studies. Reference lists of key literature reviews that examine the prevalence or risk of domestic violence among people with mental disorders will also be searched to identify potentially relevant studies. Hand searching of key journals will be carried out.

In order to extract relevant data from the extensive literature on victimisation in general among people with mental disorders, we will examine studies identified in recent systematic reviews of violence victimisation amongst people with mental disorders. This will enable us to identify studies which (may) have collected data on the prevalence and/or risk of domestic violence victimisation amongst men and women with a mental disorder; if data are not presented separately by nature of perpetrator in the original manuscript, we will contact the authors to obtain the raw data for inclusion of relevant data and summary statistics in this review. An update of these reviews will also be conducted by re-running the literature searches for the period between the upper date limits of their review and this review.

6.2.1 Electronic Databases

The following databases will be searched:

Biomedical sciences databases:

- Cochrane
- MEDLINE
- EMBASE
- Web of Knowledge
- Web of Science
- MIDIRS
- PsycINFO
- Health Management Information Consortium (HMIC)
- British Nursing Index (BNID)
- CINAHL
- Science Direct
- Academic Search Premier

Social sciences databases:

- Applied Social Sciences Index and Abstracts
- International Bibliography of the Social Sciences (IBSS)
- JSTOR
- Sociological Abstracts
- SSCI
- SIGLE
- Australasian Digital Thesis Program
- DART-Europe E Theses Portal
- ETHOS
- Networked Digital Library of Theses and Dissertations

6.2.2 Hand Searching

Hand searches of the following journals will be conducted:

- Trauma, Violence and Abuse
- Journal of Traumatic Stress
- Violence Against Women

No lower date limits have been placed on hand searches

6.2.3 Contacting Key Experts

The reviewers will contact the corresponding authors of included papers, asking them for further information about study methodology (if required) and to nominate missing studies and/or datasets. Reviewers will also contact key experts in the field of domestic violence research (eg Claudia Garcia-Moreno; Kelsey Hegarty; Jacqueline Golding; Jacqueline Campbell) and ask them to identify relevant studies. Key experts researching the area of violence victimisation in people with mental disorders (eg Tom Fahy; Kimberlie Dean; Linda Teplin; Elizabeth Walsh, Jackie Barron) will also be contacted and asked to nominate key references for inclusion.

**6.3 Bias**

6.3.1 Publication/Language and Location Bias

Easterbrook et al’s analysis of publication bias in clinical research identified that studies reporting statistically significant results were more likely to be published than those reporting no significance. Studies reporting significant results were also found to result in a greater number of publications in high citation impact journals. The authors found that publication bias was greater among observational studies than randomised controlled trials and also existed in cohort studies (Easterbrook et al., 1991).

Egger and Smith identified publication bias in relation to study location, as research conducted in less developed countries was less commonly published in a journal indexed in a literature database than research conducted in developed countries. The authors also found that studies reporting significant results were more likely to be published in English, to be cited more frequently and to receive multiple publications (Egger & Smith, 1998).

In order to address some of the bias issues outlined above, reviewers will attempt to identify theses/dissertations, reports and peer-reviewed papers based on book chapters that have been published or that are currently in press. Our decision to include studies written in languages other than English may also help to address publication bias. Furthermore, the search strategy for this review attempts to minimise the effects of location bias by searching a varied selection of electronic databases and by contacting key experts in the field to identify journal articles that may not have been indexed by the electronic databases used. As part of the analysis, the reviewers will assess to what extent the review is affected by publication bias.

6.3.2 Duplication Bias

Where the review identifies multiple eligible papers from the same study only the main paper with the total N with data of relevance to the objectives of the review will be extracted and included in the analysis.

**7. Conducting the Review**

**7.1 Title and Abstract Screening**

Literature searches of the databases listed in section 6 will be conducted and the resulting citations will be downloaded to EndNote© software, where duplicate citations will be removed. Additional citations that have been identified by forward and backward citation tracking, hand searches, key experts, and the re-examination and updating of systematic reviews will also be added to the EndNote© database.

Based on the criteria described in section 5 titles and abstracts of all downloaded citations, including those identified from the victimisation reviews, will be evaluated independently by two reviewers for a decision on inclusion or exclusion.

Where it is not possible to tell if a citation is relevant it will be included at this stage. If there is disagreement between reviewers about the citation it will also be included at this stage. Full hard copies of the papers, identified at this stage as potentially included, will be obtained.

**7.2 Retrieval and Screening of Full Text Articles**

Two reviewers will independently read the obtained papers, using a standardised checklist to assess eligibility and make a decision on inclusion (see appendix B). If there is a disagreement between the two reviewers, about the papers eligibility, LH will advise. Excluded references will be retained in separate folders within EndNote©, categorised according to the primary reason for exclusion.

In relation to the papers retrieved from the re-examination and update of the victimisation literature reviews listed in section 6.2.4, this stage will include consideration of whether the studies collected data on the prevalence and/or risk of domestic violence victimisation (i.e., whether they collected data on the *perpetrator* of the reported violence). If primary studies have collected information on domestic violence victimisation but have not presented it, the reviewers will contact the corresponding authors of the studies to request further information and/or raw data for analysis. Reviewers will also contact corresponding authors when it is not clear whether the study collected data on domestic violence victimisation.

**7.3 Data Extraction**

Data will be extracted from all studies, which have been selected for inclusion in the review using a standardised extraction form. Reviewers will also make note of the reasons for exclusion of identified studies, so that this can be summarised in an excluded studies table.

The data extraction form requires the reporting of bibliographic information, study design characteristics, study sample, and study outcomes. The study outcomes of interest include the risk and prevalence of adult lifetime and/or past year experiences of domestic violence among people with mental disorders. Details of resource use, type and severity of violence, sex-specific and age-specific prevalence rates and chronicity of mental disorders will also be extracted, if reported.

**7.4 Study Appraisal**

Studies will be methodologically appraised by two reviewers using criteria adapted from previously validated tools, which includes items assessing study design, representativeness of study samples, outcomes measured and appropriate use of statistical analysis (see appendix C). Adaptations to a critical appraisal tool for prevalence studies, developed by Loney et al (2000), included incorporating a number of sources on study methodology from the Critical Appraisal Skills Programme checklists, sources on confounding and attrition from Downs and Black 1998 and quality rating of diagnosis ascertainment by Saha et al 2005 (Downs & Black, 1998; Loney & Chambers, 2000; Saha et al., 2005). Overall study quality will be assessed according to the percentage of the maximum possible quality score attained.

The quality appraisal form has 21 questions about study quality, organised into 16 domains.  Papers receive a grade of between 0-2 for each question, giving a maximum score for this section of 42. A study is awarded 0 points if it does not meet the criteria or answer the question, 1 point if it partially meets the criteria or gives a partially satisfactory answer to the question, and 2 points if it fully meets the criteria or gives a fully satisfactory answer to the question. The overall study quality (expressed as the percentage of the maximum total score attained) will be reported for all included studies.

**7.5 Data Analysis**

The extracted data will be analysed in STATA. Basic descriptive analysis will be conducted to summarise information about the study population (e.g., the country studied, the age range studied, the sex of participants, and the type of population covered); the sample characteristics (e.g. the sample size and the response rate); and the method of case ascertainment and diagnosis (i.e., diagnostic interview, assessment of medical records).

Analysis of the prevalence (adult lifetime or past year) and risk of domestic violence victimisation will be conducted separately by gender and mental disorder (including chronicity of disorder, if applicable). The risk of victimisation will be reported primarily using odds ratios, but where sufficient data are available, population attributable risk will also be calculated. 95% confidence intervals will be reported for all measures of prevalence, odds ratios and relative risk (i.e. comparing domestic violence victimisation between people who have a mental disorder and appropriate controls). The results of the analyses will be tabulated and/or displayed graphically using forest plots. Odds ratios will also be used to create funnel plots in order to detect publication bias.

Graphical methods and the calculation of Cochrane’s I² statistic will assist the analysis of the heterogeneity of the included studies. After considering study heterogeneity extracted summary statistics will be pooled, if appropriate, to determine an overall prevalence and risk of domestic violence victimisation amongst people with specific mental disorders. If data are pooled, the overall summary statistics (measures of prevalence and odds ratios, with 95% confidence intervals) will also be presented.

A critical conceptual analysis of included papers will also be conducted, examining how domestic violence is measured and conceptualised (including type and severity of violence) across studies and the potential effect this may have on reported outcomes.

**References**

Brown, G. W., Harris, T. O., Hepworth, C., & Robinson, R. (1994). Clinical and Psychosocial Origins of Chronic Depressive Episodes II: A Patient Enquiry. *British Journal of Psychiatry, 165,* 457-465.

Campbell, J. C. (2002). Health consequences of intimate partner violence. *The Lancet, 359,* 1331-1336.

Dalsbo, T. K. & Johme, T. (2006). "Cognitive behavioural therapy for men who physically abuse their female partner (Protocol) ". *Cochrane Database of Systematic Reviews*.

Downs, S. H. & Black, N. (1998). "The feasibility of creating a checklist for the assessment of the methodological quality both of randomised and non-randomised studies of health care interventions.". *J Epidemiol Comm Health, 52,* 377-384.

Easterbrook, P. J., Berlin, J. A., Gopalan, R., & Matthews, D. R. (1991). "Publication bias in clinical research.". *The Lancet, 337,* 867-872.

Egger, M. & Smith, G. D. (1998). "Meta-analysis bias in location and selection of studies.". *BMJ, 316,* 61.

Friedman, S. H. & Loue, S. (2007). Incidence and prevalence of intimate partner violence by and against women with severe mental illness. *Journal of Women's Health, 16,* 471-480.

Golding, M. J. (1999). Intimate Partner Violence as a Risk Factor for Mental Disorders: A Meta-Analysis. *Journal of Family Violence, 14,* 99-132.

Goodman, L. A., Salyers, M. P., Mueser, K. T., Rosenberg, D. S., Swartz, M., Essock, M. S. et al. (2001). Recent victimization in women and men with severe mental illness: prevalence and correlates. *Journal of Traumatic Stress, 14,* 615-632.

Howard, L., Trevillion, K., Khalifeh, H., Woodall, A., Agnew-Davies, R., & Feder, G. (2010). Domestic Violence and Severe Psychiatric Disorders: prevalence and interventions. *Psychological Medicine, Nov 6,* 1-13.

Loney, P. L. & Chambers, L. W. (2000). "Critical appraisal of the health research literature: prevalence or incidence of a health problem.". *Chronic Dis Canada, 19,* 170-177.

McGuffin, P., Farmer, A. E., & Harvey, I. (1991). A polydiagnostic application of operational criteria in studies of psychotic illness. Development and reliability of the OPCRIT system. *Archives ofGeneral Psychiatry, 48,* 764-770.

NICE (2008). *The guidelines manual.* London: National Institute for Health and Clinical Excellence.

Ramsay, J., Richardson, J., Carter, Y. H., Davidson, L. L., & Feder, G. (2002). Should health professionals screen for domestic violence? Systematic review. *British Medical Journal, 325,* 1-13.

Saha, S., Chant, D., Welham, J., & McGrath, J. (2005). "A systematic review of the prevalence of schizophrenia.". *PLoS Med, 2,* 141.

Teplin, L. A., McClelland, G. M., & ram, K. M. e. a. (2005). Crime victimization in adults with severe mental illness - Comparison with the national crime victimization survey. *Archives of General Psychiatry, 62,* 911-921.

Walby, S. (2004). *The Cost of Domestic Violence* London: Women and Equality Unit/DTI.

Wing, J. K. (1994). The Schedules for Clinical Assessment in Neuropsychiatry. Geneva, World Health Organization-Division of Mental Health .

**Appendix A**

**Search Terms**

Search terms for use in the Ovid Platform (Medline, Embase, PsychInfo, British Nursing Index, Health Management Information Consortium and IBSS)

1. Domestic violence/
2. Family violence/
3. Partner abuse/
4. Partner violence/
5. Spouse abuse/
6. Battered women/
7. ((abus$ OR batter$ OR violen$ OR beat$) adj2 (domestic OR partner$ OR family OR families OR spouse OR woman OR women OR men OR man OR female$ OR male$ OR wife OR wives OR husband$ OR boyfriend$ OR girlfriend$ OR elder$ OR brother$ OR sister$ OR father$ OR mother$ OR daughter$ OR son$ OR carer$).mp.)
8. (domestic adj5 homicid$).mp
9. 1 OR 2 OR 3 OR 4 OR 5 OR 6 OR 7 OR 8
10. Mental disorder/
11. Mental illness/
12. Mental health/
13. Mentally ill persons/
14. (Mental$ adj2 (problem$ OR difficult$ OR disorder$ OR ill$ OR health).mp.)
15. Mental health services/
16. Community Mental Health Services/
17. ((mental OR psychiatr$ OR psycholog$) adj2 (inpatient$ OR outpatient$ OR hospital$ OR clinic$ OR service$ OR ward$ OR healthcare).mp)
18. Schiz$
19. Psychosis
20. Psychotic
21. Bipolar
22. Depress$
23. Mania OR manic
24. Neurosis OR psychoneurosis
25. Obsessive OR compulsive
26. Personality disorder/ OR anankastic personality disorder/ OR antisocial personality disorder/ OR avoidant personality disorder/ OR borderline personality disorder/ OR compulsive personality disorder/ OR dependent personality disorder/ OR histrionic personality disorder/ OR narcissistic personality disorder/ OR obsessive compulsive personality disorder/ OR paranoid personality disorder/ OR passive-aggressive personality disorder/ OR schizoid personality disorder/ OR schizotypal personality disorder/ OR ((anankastic OR asocial OR antisocial OR avoidant OR borderline OR dependent OR dissocial OR histrionic OR narcissistic OR obsessive OR compulsive OR paranoid OR passive-aggressive OR psychopath$ OR sadist$ OR sadomasochistic OR schizo$ OR sociopath$) adj person$).tw. OR (personality AND disorder$r) OR psychopath$.tw OR sociopath$.tw
27. Eating disorders/ OR Anorexia Nervosa/ OR Binge-Eating Disorder/or Bulimia Nervosa/ OR ((anorexi$ OR bulimi$) AND nervosa) OR eating disorder$ OR binge-eat$ OR (bing$ adj eat$) OR (compulsive adj (eat$ or vomit$ or purg$))
28. ((Delusional OR paranoi$ OR mood OR neurotic OR stress OR reactive OR combat OR somatoform OR somatization OR somatisation OR anxiety OR phobic OR obsessive-compulsive OR adjustment OR dissociat$) adj2 disorder$)
29. 10 OR 11 OR 12 OR 13 OR 14 OR 15 OR 16 OR 17 OR 18 OR 19 OR 20 OR 21 OR 22 OR 23 OR 24 OR 25 OR 26 OR 27 OR 28
30. 9 AND 29

**Appendix B**

**Prevalence and Risk of Domestic Violence Victimisation amongst People with Mental Disorders: Checklist**

Study Type:

|  |
| --- |

Author Name:

|  |
| --- |

Paper title:

|  |
| --- |

Reviewer ID:

|  |
| --- |

**CHECKLIST**

Does the paper meet **each** of the following inclusion criteria?

| **Inclusion criteria** | **If yes tick box** |
| --- | --- |
| Study is published in a peer-reviewed journal, report, or is a thesis/dissertation. |  |
| Study uses an eligible study design (randomised controlled trial, non-randomised controlled trial, parallel group study, before and after study, interrupted time series, cohort study, case-control study, cross-sectional study) |  |
| Sample includes participants aged 16 years or older |  |
| Sample includes participants who have experienced adult lifetime and/or past year domestic violence victimisation |  |
| Sample includes participants with a mental disorder (as measured using a validated assessment) |  |
| Study results include the prevalence, incidence, risk or odds of domestic violence victimisation, or presents data from which these statistics can be calculated |  |

If the paper does not meet **all** of the above criteria, please indicate below the reasons why:

| **Exclusion criteria** | **If yes tick box** |
| --- | --- |
| Study is published in as a book, conference paper, dissertation/thesis, general comment paper, letter, editorial or other non-peer reviewed format. |  |
| Study uses an ineligible study design (single case study, qualitative interview, focus group interviews) |  |
| Sample is aged 15 or younger (or includes participants aged 15 or younger and does not provide appropriately disaggregated data) |  |
| Sample does not include participants with a mental disorder (or includes participants with mental disorder but does not provide appropriately disaggregated data) |  |
| Study does not measure adult lifetime or past year domestic violence victimisation |  |
| Study results do not include the prevalence, incidence, risk or odds of domestic violence victimisation and the study does not present data from which these statistics can be calculated |  |

If the paper meets any of the exclusion criteria do not proceed any further.

**Appendix C**

**Quality Appraisal Form**

**Please complete part 1 for all study designs and complete the relevant sections for part 2, specific to study design.**

Score the answer to each question by ticking 0, 1 or 2:

0 – study does not meet criteria/answer question

1 – Study partially meets criteria/gives a partially satisfactory answer to the question

2 – Study fully meets criteria/gives a fully satisfactory answer to the question

| **Part 1** | | | | | | | | | |
| --- | --- | --- | --- | --- | --- | --- | --- | --- | --- |
| **Screening questions** | | | | | | **Score** | | | |
|  | Question | | Comments | | | 0 | | 1 | 2 |
| 1 | Did the study ask a clearly focused question?  *– Is the hypothesis/aim/objective of the study clearly described?*  *-Is the study question focused in terms of the outcomes considered?* | |  | | |  | |  |  |
| 2 | Is the study design appropriate for the research question? | |  | | |  | |  |  |
| 3 | Was a validated tool used to assess mental disorder?   - - *Validated diagnostic instrument used (i.e., in diagnostic interview or case file assessment) =2*   - *Validated screening instrument used (i.e., in screening interview or case file assessment) =1* | |  | | |  | |  |  |
| **Continue only if score on each of questions 1 and 2 is one or more** | | | | | | | | | |
| **Detailed questions** | | | | | | | | | |
| **Measurement of risk of selection bias** | | | | | | | | | |
| 4a | | Is the sampling method appropriate for the research question?  *Consider:*  *-The sampling method used (i.e. random selection of subjects)*  *- If applicable, is there appropriate selection of controls?* | |  |  | |  | |  |
| 4b | | Are subjects appropriately defined?  *Consider:*  *- Inclusion/ exclusion criteria specified*  *- Inclusion/exclusion criteria appropriate* | |  |  | |  | |  |
| 4c | | Is the sample size appropriate?  *Consider:*  *- Is the sample size justified?*  *- Were a sufficient number of cases selected?*  *- If applicable, were a sufficient number of controls selected?* | |  |  | |  | |  |
| 4d | | Is the study sample representative of the population of interest?  *-Do the authors assess the representativeness of the study sample?* | |  |  | |  | |  |
| 4e | | Does the level of non-participation risk introducing bias?  *Consider:*  *-Are key demographic characteristics of non-participants reported and compared against participants?*  *-Does the study report on the impact of non-participation?*  *-If applicable, rates of attrition reported* | |  |  | |  | |  |
| 5 | | Is the study setting appropriate to the aims of the research? (e.g. setting, location, relevant dates) | |  |  | |  | |  |
| 6 | | Is the method of data collection appropriate for the aims of the research? | |  |  | |  | |  |
| **Measurement of risk of reporting bias** | | | | | | | | | |
| 7 | | Are suitable/standard criteria used for measurement of domestic violence?  *Consider:*  *-Criteria of domestic violence was clearly defined*  *-Potential for bias of measurement*  *-If measures piloted*  *- Standardised/pre-validated measures (score 2 points)*  *- Researchers developed their own measure (score 1 point)*  *- No details of measurement were provided (score 0 point)* | |  |  | |  | |  |
| 8 | | Are known confounders accounted for by study design?  *- Was consideration of confounding factors accounted for in study design?* | |  |  | |  | |  |
| 9 | | Are known confounders accounted for in the analyses? | |  |  | |  | |  |
| 10 | | Are the statistical tests used to assess the main outcomes appropriate?  *-Was there adequate adjustment for confounding in the analyses?*  *- Do the analyses adjust for different lengths of follow-up (if applicable)?* | |  |  | |  | |  |
| 11a | | Are the estimates reported with confidence intervals and in detail by sub-group (if appropriate)?  *- Were the findings reported clearly?* | |  |  | |  | |  |
| 11b | | Are statistically non-significant results presented? | |  |  | |  | |  |
| 11c | | Are data for relevant variables complete? | |  |  | |  | |  |
| 12 | | Was the conduct of the fieldwork appropriate to the study setting?  *-Was the allocation of the interviewer/interpreter sensitive to the background of the participant?*  *-Were fieldworkers trained and supported to work with people who have experienced domestic violence?* | |  |  | |  | |  |
| 13 | | Were ethical considerations appropriately considered?  -*Did researchers obtain informed consent from all participants?*  *- Did researchers take adequate precautions to safeguard participant anonymity and confidentiality?*  *-Did fieldworkers offer information about domestic violence support and referral options to all participants?*  -*Were fieldworkers appropriately trained to deal with participant distress?* | |  |  | |  | |  |
| 14 | | Do the findings support the conclusions? | |  |  | |  | |  |
| 15 | | Are the strengths and weaknesses of the research discussed? | |  |  | |  | |  |

Calculate total score (out of a possible total of 42):
