# Supplementary material for: Domestic Violence and Perinatal Mental Disorders: A Systematic Review and Meta-Analysis
Source: PLoS Med. 2013 May 28;10(5):e1001452. doi: 10.1371/journal.pmed.1001452 (PMC3665851; doi:10.1371/journal.pmed.1001452)
Supplement: Text S3 — Search terms for Medline, Embase, and PsycINFO. (DOC) [file pmed.1001452.s006.doc]

**Text S3: Search Terms for MEDLINE, EMBASE, PsycInfo**

1. Domestic violence/
2. Family violence/
3. Partner abuse/
4. Partner violence/
5. Spouse abuse/
6. Battered women/
7. ((abus$ OR batter$ OR violen$ OR beat$) adj2 (domestic OR partner$ OR family OR families OR spouse OR woman OR women OR men OR man OR female$ OR male$ OR wife OR wives OR husband$ OR boyfriend$ OR girlfriend$ OR elder$ OR brother$ OR sister$ OR father$ OR mother$ OR daughter$ OR son$ OR carer$).mp.)
8. (domestic adj5 homicid$).mp
9. 1 OR 2 OR 3 OR 4 OR 5 OR 6 OR 7 OR 8
10. Mental disorder/
11. Mental illness/
12. Mental health/
13. Mentally ill persons/
14. (Mental$ adj2 (problem$ OR difficult$ OR disorder$ OR ill$ OR health).mp.)
15. Mental health services/
16. Community Mental Health Services/
17. ((mental OR psychiatr$ OR psycholog$) adj2 (inpatient$ OR outpatient$ OR hospital$ OR clinic$ OR service$ OR ward$ OR healthcare).mp)
18. Schiz$
19. Psychosis
20. Psychotic
21. Bipolar
22. Depress$
23. Mania OR manic
24. Neurosis OR psychoneurosis
25. Obsessive OR compulsive
26. Personality disorder/ OR anankastic personality disorder/ OR antisocial personality disorder/ OR avoidant personality disorder/ OR borderline personality disorder/ OR compulsive personality disorder/ OR dependent personality disorder/ OR histrionic personality disorder/ OR narcissistic personality disorder/ OR obsessive compulsive personality disorder/ OR paranoid personality disorder/ OR passive-aggressive personality disorder/ OR schizoid personality disorder/ OR schizotypal personality disorder/ OR ((anankastic OR asocial OR antisocial OR avoidant OR borderline OR dependent OR dissocial OR histrionic OR narcissistic OR obsessive OR compulsive OR paranoid OR passive-aggressive OR psychopath$ OR sadist$ OR sadomasochistic OR schizo$ OR sociopath$) adj person$).tw. OR (personality AND disorder$r) OR psychopath$.tw OR sociopath$.tw
27. Eating disorders/ OR Anorexia Nervosa/ OR Binge-Eating Disorder/or Bulimia Nervosa/ OR ((anorexi$ OR bulimi$) AND nervosa) OR eating disorder$ OR binge-eat$ OR (bing$ adj eat$) OR (compulsive adj (eat$ or vomit$ or purg$))
28. ((Delusional OR paranoi$ OR mood OR neurotic OR stress OR reactive OR combat OR somatoform OR somatization OR somatisation OR anxiety OR phobic OR obsessive-compulsive OR adjustment OR dissociat$) adj2 disorder$)
29. 10 OR 11 OR 12 OR 13 OR 14 OR 15 OR 16 OR 17 OR 18 OR 19 OR 20 OR 21 OR 22 OR 23 OR 24 OR 25 OR 26 OR 27 OR 28
30. 9 AND 29
