# Supplementary material for: Domestic Violence and Perinatal Mental Disorders: A Systematic Review and Meta-Analysis
Source: PLoS Med. 2013 May 28;10(5):e1001452. doi: 10.1371/journal.pmed.1001452 (PMC3665851; doi:10.1371/journal.pmed.1001452)
Supplement: Text S4 — Critical appraisal checklist for included studies. (DOC) [file pmed.1001452.s007.doc]

**Text S4: Critical Appraisal Checklist for Included Studies**

Each question is scored as follows:

0 – study does not meet criteria/answer question

1 – Study partially meets criteria/gives a partially satisfactory answer to the question

2 – Study fully meets criteria/gives a fully satisfactory answer to the question

| **Part 1** | | | | | |
| --- | --- | --- | --- | --- | --- |
| **Questions** | | | **Score** | | |
|  | Question | Comments | 0 | 1 | 2 |
| 1 | Did the study ask a clearly focused question?  *– Is the hypothesis/aim/objective of the study clearly described?*  *-Is the study question focused in terms of the outcomes considered?* |  |  |  |  |
| 2 | Is the study design appropriate for the research question? |  |  |  |  |
| 3 | Was a validated tool used to assess mental disorder?   - - *Validated diagnostic instrument used (i.e., in diagnostic interview or case file assessment) =2*   - *Validated screening instrument used (i.e., in screening interview or case file assessment) =1* |  |  |  |  |
| 4a | Is the sampling method appropriate for the research question?  *Consider:*  *-The sampling method used (i.e. random selection of subjects)*  *- If applicable, is there appropriate selection of controls?* |  |  |  |  |
| 4b | Are subjects appropriately defined?  *Consider:*  *- Inclusion/ exclusion criteria specified*  *- Inclusion/exclusion criteria appropriate* |  |  |  |  |
| 4c | Is the sample size appropriate?  *Consider:*  *- Is the sample size justified?*  *- Were a sufficient number of cases selected?*  *- If applicable, were a sufficient number of controls selected?* |  |  |  |  |
| 4d | Is the study sample representative of the population of interest?  *-Do the authors assess the representativeness of the study sample?* |  |  |  |  |
| 4e | Does the level of non-participation risk introducing bias?  *Consider:*  *-Are key demographic characteristics of non-participants reported and compared against participants?*  *-Does the study report on the impact of non-participation?*  *-If applicable, rates of attrition reported* |  |  |  |  |
| 5 | Is the study setting appropriate to the aims of the research? (e.g. setting, location, relevant dates) |  |  |  |  |
| 6 | Is the method of data collection appropriate for the aims of the research? |  |  |  |  |
| 7 | Are suitable/standard criteria used for measurement of domestic violence?  *Consider:*  *-Criteria of domestic violence was clearly defined*  *-Potential for bias of measurement*  *-If measures piloted*  *- Standardised/pre-validated measures (score 2 points)*  *- Researchers developed their own measure (score 1 point)*  *- No details of measurement were provided (score 0 point)* |  |  |  |  |
| 8 | Are known confounders accounted for by study design?  *- Was consideration of confounding factors accounted for in study design?* |  |  |  |  |
| 9 | Are known confounders accounted for in the analyses? |  |  |  |  |
| 10 | Are the statistical tests used to assess the main outcomes appropriate?  *-Was there adequate adjustment for confounding in the analyses?*  *- Do the analyses adjust for different lengths of follow-up (if applicable)?* |  |  |  |  |
| 11a | Are the estimates reported with confidence intervals and in detail by sub-group (if appropriate)?  *- Were the findings reported clearly?* |  |  |  |  |
| 11b | Are statistically non-significant results presented? |  |  |  |  |
| 11c | Are data for relevant variables complete? |  |  |  |  |
| 12 | Was the conduct of the fieldwork appropriate to the study setting?  *-Was the allocation of the interviewer/interpreter sensitive to the background of the participant?*  *-Were fieldworkers trained and supported to work with people who have experienced domestic violence?* |  |  |  |  |
| 13 | Were ethical considerations appropriately considered?  -*Did researchers obtain informed consent from all participants?*  *- Did researchers take adequate precautions to safeguard participant anonymity and confidentiality?*  *-Did fieldworkers offer information about domestic violence support and referral options to all participants?*  -*Were fieldworkers appropriately trained to deal with participant distress?* |  |  |  |  |
| 14 | Do the findings support the conclusions? |  |  |  |  |
| 15 | Are the strengths and weaknesses of the research discussed? |  |  |  |  |

Calculate total score (out of a possible total of 42)
